# RavA‐ViaA antibiotic response is linked to Cpx and Zra2 envelope stress systems in *Vibrio cholerae*

**DOI:** 10.1128/spectrum.01730-23

**Published:** 2023-10-20

**Authors:** Evelyne Krin, André Carvalho, Manon Lang, Anamaria Babosan, Didier Mazel, Zeynep Baharoglu

**Affiliations:** 1 Institut Pasteur, Université Paris Cité, CNRS UMR3525, Unité Plasticité du Génome Bactérien, Paris, France; 2 Sorbonne Université, Collège doctoral, Paris, France; Indian Institute of Science Bangalore, Bangalore, Karnataka, India

**Keywords:** antibiotic resistance, antibiotic tolerance, ravA viaA, bacterial membrane, *Vibrio cholerae*, sub-MIC antibiotics

## Abstract

RavA-ViaA were reported to play a role in aminoglycoside (AG) sensitivity, but the mechanisms remain elusive. Here, we performed competition and survival experiments to confirm that deletion of *ravA-viaA* increases tolerance of the Gram-negative pathogen *Vibrio cholerae* to low and high AG concentrations during aerobic growth. Using high-throughput strategies in this species, we identify Cpx and Zra2 two-component systems as new partners of RavA-ViaA. We show that the AG tolerance of *∆ravvia* requires the presence of these membrane stress sensing two-component systems. We propose that deletion of the RavA-ViaA function facilitates the response AGs because of a pre-activated state of Cpx and Zra2 membrane stress response systems. We also find an impact of these genes on vancomycin resistance, and we show that simultaneous inactivation of *ravvia* function together with envelope stress response systems leads to outer membrane permeabilization. Vancomycin is mostly used for Gram-positive because of its low efficiency for crossing the Gram-negative outer membrane. Targeting of the *ravA-viaA* operon for inactivation could be a future strategy to allow uptake of vancomycin into multidrug-resistant Gram-negative bacteria.

**IMPORTANCE:**

The RavA-ViaA complex was previously found to sensitize *Escherichia coli* to aminoglycosides (AGs) in anaerobic conditions, but the mechanism is unknown. AGs are antibiotics known for their high efficiency against Gram-negative bacteria. In order to elucidate how the expression of the *ravA-viaA* genes increases bacterial susceptibility to aminoglycosides, we aimed at identifying partner functions necessary for increased tolerance in the absence of RavA-ViaA, in *Vibrio cholerae*. We show that membrane stress response systems Cpx and Zra2 are required in the absence of RavA-ViaA, for the tolerance to AGs and for outer membrane integrity. In the absence of these systems, the *∆ravvia* strain’s membrane becomes permeable to external agents such as the antibiotic vancomycin.

## INTRODUCTION

Antibiotic resistance is a growing public health problem. Common resistance mechanisms developed by bacteria consist of limiting antibiotic entry, increasing efflux, degrading the antibiotic, or mutating the target. Decreasing intracellular concentrations of antibiotics is one of the most frequent resistance strategies. The aminoglycoside (AG) class of antibiotics targets the ribosome, leading to mistranslation, protein misfolding, and eventually cell death. AGs are highly efficient against Gram-negative bacteria (1). They comprise kanamycin, tobramycin, gentamicin, neomycin, amikacin, and streptomycin and are commonly used worldwide. The currently accepted model for AG entry (2, 3) starts with the PMF- (4 – 6) and respiration-dependent (7 – 9) entry of a small AG quantity, leading to mistranslation by the ribosome. Incorporation of mistranslated proteins would then lead to membrane damage and a subsequent second step AG uptake in large amounts (6, 10).

The *ravA-viaA* operon (formerly *yieMN*) was found to sensitize Gram-negative bacteria to the AGs (11), but the mechanism remains elusive. The presence of RavA-ViaA has been reported mostly in ɣ-proteobacteria. Notably, *ravA-viaA* were found in 37 out of 50 randomly chosen enterobacteria (12).

RavA (regulatory ATPase variant A) belongs to the class of P-loop AAA+ proteins, which hydrolyze ATP and are involved in diverse molecular processes such as protein degradation, assembly of membrane complexes, DNA repair, and others. ViaA (VWA interacting with AAA+ ATPase) carries a von Willebrand factor type A (VWA) domain and interacts with RavA and stimulates its ATPase activity (13). Structural work revealed that RavA and ViaA interact with each other (13) and form a complex with a third partner, LdcI (or CadA) (12, 13), leading to a cage-like structure up to 1 MDa in size (14). It was also shown that the RavA-ViaA complex interacts with phospholipids at the inner membrane (15).

The involvement of *ravA-viaA* genes in AG susceptibility was originally identified by independent approaches in different proteobacteria*, Escherichia coli* (11) and *Vibrio cholerae* (16). In *E. coli,* overexpression of *ravA-viaA* sensitizes to gentamicin, and its deletion was shown to increase AG resistance (11, 17) but only in anaerobic conditions and low energy state (15, 18). In *V. cholerae*, the *ravA-viaA* operon (VC_A0762-VC_A0763) is also involved in the response to low doses (below MIC, or sub-MIC) of AGs, this time in aerobic conditions. While studying the response of *V. cholerae* to sub-MIC tobramycin, we have conducted a high-throughput transposon insertion sequencing (TN-seq) screen (19, 20) where the most enriched insertions were detected in the VC_A0762 (*viaA*) and VC_A0763 (*ravA*) genes (respectively, 60× and 30× enrichment), suggesting that inactivation of the operon confers a growth advantage in the presence of AGs in *V. cholerae*. This is consistent with our previous results showing that genotoxic stress induced by tobramycin is limited in the absence of this operon (16).

The PMF is involved in the first phase of AG uptake (21), while the second phase occurs in response to mistranslation (or through sugar transporters). PMF is produced by the activity of electron transfer chains in respiratory complexes, where Fe-S cluster are key actors. It was shown that Fe-S biogenesis and their fueling to respiratory complexes have a direct impact on AG uptake (22). Previous studies reported that RavA and ViaA interact with Fe-S cluster biogenesis machineries and also with components of the major Fe-S cluster containing Nuo respiratory complex (11, 23), suggesting that RavA-ViaA could contribute to folding of the respiratory complex I. Therefore, it was proposed that RavA and ViaA sensitize *E. coli* to AGs by facilitating Fe-S targeting to complex I and as a consequence an increased level of PMF. Note that *E. coli* and *V. cholerae* are very dissimilar in respect to the respiration complexes, Fe-S biogenesis, and oxidative stress response pathways. For instance, *V. cholerae* lacks the above-mentioned Cyo/Nuo complex I and lacks also the SUF Fe-S biogenesis system used under oxidative stress. RavA-ViaA complex was, thus, proposed to allow AGs to accumulate inside the cells, presumably by enhancing their uptake of AGs (18).

In order to shed light into how these genes modulate bacterial susceptibility to AGs, it’s important to understand in which conditions these genes are expressed, which bacterial functions are necessary for AG sensitization, and which bacterial processes are affected by these genes.

Here, we first extensively confirmed the RavA-ViaA-dependent AG susceptibility and tolerance phenotypes in *V. cholerae*. Next, high-throughput approaches identified the involvement of envelope stress responses in these phenotypes. TN-seq showed that inactivation of *cpxP* repressor*,* i.e., activation Cpx response, is beneficial in *∆ravvia*. Cpx responds to conditions that cause misfolding of inner membrane (IM) and periplasmic proteins, and subsequent membrane defect [for review, see reference (24)]. Cpx also down-regulates outer membrane proteins. In parallel, transcriptomic data in *∆ravvia* showed strong induction of VC_1314 and the VC_1315-VC_1316 operon which presents similarities with the Cpx and Zra two-component envelope stress response systems. In *E. coli*, ZraSR contributes to antibiotic resistance and is important for membrane integrity (25). *∆zra* has increased membrane disruption during treatment with membrane-targeting antibiotics. Zra chaperone activity is enhanced in the presence of zinc ions. In this case, zinc was proposed to be a marker of envelope stress perturbation where ZraPSR is a sentinel sensing and responding to zinc entry into the periplasm (26).

We find that the AG tolerance conferred by *ravA-viaA* deletion in *V. cholerae* requires the presence of the Cpx system and Zra-like system, which we propose to name *zraP2-zraS2-zraR2*. We further show for the first time that *ravA-viaA* deletion together with inactivation of Cpx or Zra2 envelope stress responses leads to outer membrane permeabilization and may confer vancomycin sensitivity to Gram-negative bacteria.

## RESULTS

### *ravvia* deletion decreases sub-MIC aminoglycoside susceptibility in *V. cholerae*

In order to evaluate AG-related roles of RavA-ViaA, we assessed the effect of *ravA-viaA* deletion (referred to as *∆ravvia* below) and overexpression (chromosomal extra-copy, referred to as WT::*ravvia*OE+, where OE stands for overexpression). We first performed competitions against *V. cholerae* WT, in the absence and presence of sub-MIC doses of AGs: tobramycin (TOB) and gentamicin (GEN), and also of antibiotics from families other than AGs: chloramphenicol (CM) that targets translation and ciprofloxacin (CIP) that targets DNA replication.

**Fig 1:**
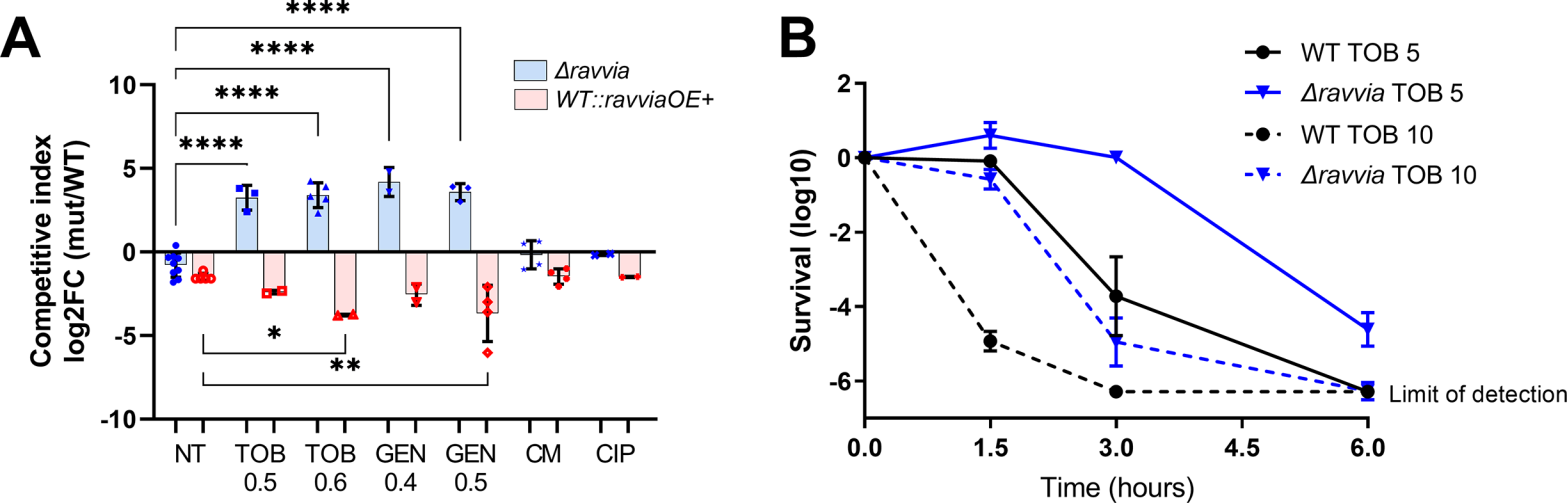
Effect of RavA-ViaA on fitness and tolerance to aminoglycosides. (**A**) Competition experiments mixing *ravvia* deletion (∆) and extra-copy (OE+) mutants and WT in indicated conditions. NT, non-treated; TOB, tobramycin; GEN, gentamicin; CM, chloramphenicol; CIP, ciprofloxacin. The *y*-axis represents log_2_ of competitive index value calculated as described in the methods. A competitive index of 1 (i.e., log_2_ value of 0) indicates equal growth of both strains. For statistical significance calculations, we used one-way ANOVA. **** means *P* < 0.0001, *** means *P* < 0.001, ** means *P* < 0.01, * means *P* < 0.05. Only significant *P* values are shown. Number of replicates for each experiment: 3 < *n* < 8. Concentrations are indicated in µg/mL. (**B**) Survival of indicated strain to lethal tobramycin treatment. *V. cholerae* WT and deletion mutant cultures were grown without antibiotics up to early exponential phase, and serial dilutions were plated on MH medium without antibiotics. Exponential phase cultures were then treated with antibiotics at lethal concentrations for the indicated times. At each time point, dilutions were spotted on MH. *Y*-axis shows survival calculated as a number of colonies at time TN divided by the initial number of colonies before antibiotic treatment. TOB: tobramycin 5 or 10 µg/mL.

Competition results show that (Fig. 1A) (i) *V. cholerae ∆ravvia* has a growth advantage compared to WT during growth with AGs TOB and GEN (40% and 50% MIC); (ii) *V. cholerae WT::ravviaOE+* has a growth disadvantage compared to WT with TOB and GEN; and (iii) no significant effect is observed in the presence of sub-MICs of tested antibiotics other than AGs. This suggests the specificity of the mechanism of action of RavA-ViaA to AGs, in *V. cholerae*. Note that the beneficial effect of *∆ravvia* is observed here during aerobic growth, while in *E. coli*, it can only be observed in anaerobic conditions.

### *ravvia* deletion increases tolerance to high doses of aminoglycosides in *V. cholerae*

Next, we tested the effect of *ravvia* deletion on resistance by measuring the minimal inhibitory concentration (MIC) and on survival to lethal treatment with AGs. The MIC of *∆ravvia* seemed unchanged or slightly increased compared to WT (1.2 to 1.5 µg/mL instead of 1.2 µg/mL) (Table 1). Single ∆*ravA* or ∆*viaA* shows the same MIC as the deletion of the whole operon, as expected, since the two proteins form a complex. The *ravviaOE+* mutant shows lower resistance (MIC at 0.75 µg/mL) (Table 1).

**TABLE 1:** MICs of tobramycin on MH-agar plates

MIC (µg/mL)	MH
∆*ravvia*	1.2–1.5
*WT::ravviaOE+*	0.75
∆*ravA*	1.2–1.5
∆*viaA*	1.2–1.5
WT	1.2

For survival to lethal treatment, we tested the tolerance of the strains to 5× and 10× MIC TOB doses for 6 h. Deletion of *ravvia* strongly increases survival to lethal TOB doses when compared to the WT strain (Fig. 1B). No effect is observed upon treatment with CIP, Trimethoprim, and Carbenicillin (Fig. S1). Treatment to TOB 5 µg/mL during 3 h shows that *∆ravvia* is not affected by TOB during this treatment window (Fig. 1B)**,** but these cells still die upon longer treatment periods (6 h) (Fig. 1B), excluding any bacteriostatic action of AGs in *∆ravvia* mutant. Thus, *∆ravvia* is killed upon lethal AG treatment, even though at a much slower rate than the WT, which is the definition of a tolerant population (27).

### The effect of *ravvia* is only partly due to differential aminoglycoside uptake

**Fig 2:**
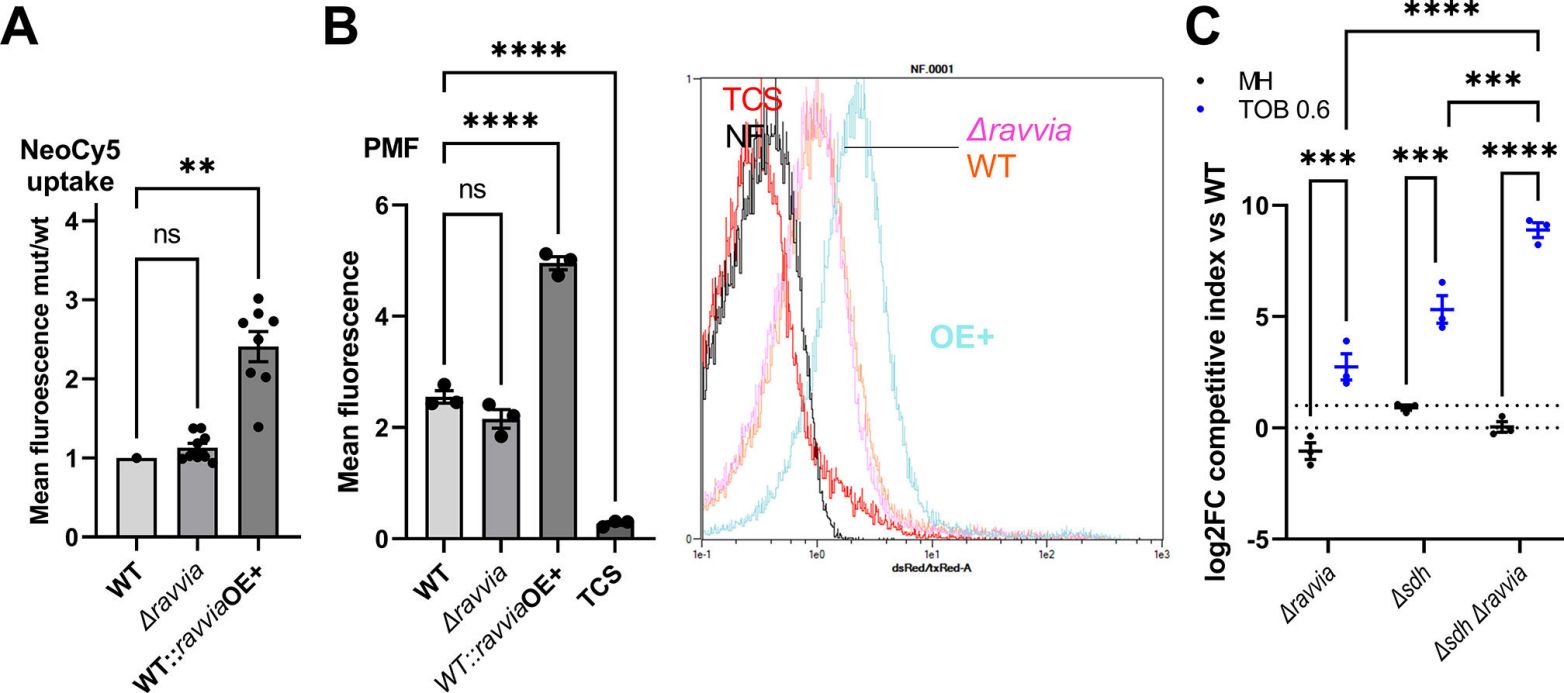
Effect of RavA-ViaA on AG uptake and membrane potential. (**A**) Intracellular level of neomycin coupled to the fluorophore Cy5 measured by fluorescence-associated flow cytometry. Error bars represent standard deviation. (**B**) Quantification of changes in PMF using Mitotracker Red fluorescence measured by flow cytometry. Representative acquisitions are shown: fluorescence is represented in the *x*-axis (FITC channel), the *y*-axis represents the number of events corresponding to the number of cells, normalized to height (same number of total cells for both conditions). Each plot represents one experiment. (**C)** Competition experiments of *V. cholerae* WT and indicated mutants. MH: no antibiotic treatment (black). TOB: tobramycin 0.6 µg/mL (blue). The *y*-axis represents log_2_ of competitive index value calculated as described in the methods. A competitive index of 1 (i.e., log_2_ value of 0) indicates equal growth of both strains. For statistical significance calculations, we used one-way ANOVA. **** means *P* < 0.0001, *** means *P* < 0.001, ** means *P* < 0.01, * means *P* < 0.05. ns, non-significant. Number of replicates for each experiment: *n* = 3. Only significant *P* values are shown.

We tested whether RavA-ViaA complex impacts AG entry, using the AG neomycin coupled to the fluorophore Cy5 (Neo-cy5), as previously done (28 – 30). Neo-cy5 is a fluorescent AG specifically designed for bacterial uptake studies that retains the properties of AGs in terms of uptake, mode of action, and activity against Gram-negative bacteria (31). In this assay, cell fluorescence is proportional to Neo-cy5 uptake. AG uptake increased in *ravvia*OE+ (Fig. 2A), suggesting that RavA-viaA overexpression facilitates AG entry into the bacterial cell. Surprisingly, AG uptake is not decreased in *V. cholerae ∆ravvia* (Fig. 2A). Since AG uptake depends on PMF, we tested the effect of *∆ravvia* on PMF. We used Mitotracker assay (29, 32), based on a fluorescent dye which accumulates inside the cell in a PMF-dependent way. We observed increased PMF in *ravvia*OE+ strain (Fig. 2B), strengthening the notion that increased AG entry of *ravvia*OE+ is due to increased PMF. Conversely, no significant decrease of PMF was detected in the AG tolerant ∆*ravvia* strain (Fig. 2B), suggesting that the effect of *ravvia* on AG susceptibility may require additional explanation as simply PMF modulation. Moreover, as expected, *sdh* (succinate dehydrogenase) deletion increased fitness in AGs because of a decrease in PMF (5). Simultaneous deletion of *sdh* and *ravvia* shows an additive fitness advantage (Fig. 2C), suggesting that the mechanisms of increased fitness of *∆ravvia* in sub-MIC TOB are not through a common pathway with the *∆sdh*-dependent PMF decrease.

### High-throughput approaches point to a role of membrane stress two-component systems in *∆ravvia*

**Fig 3:**
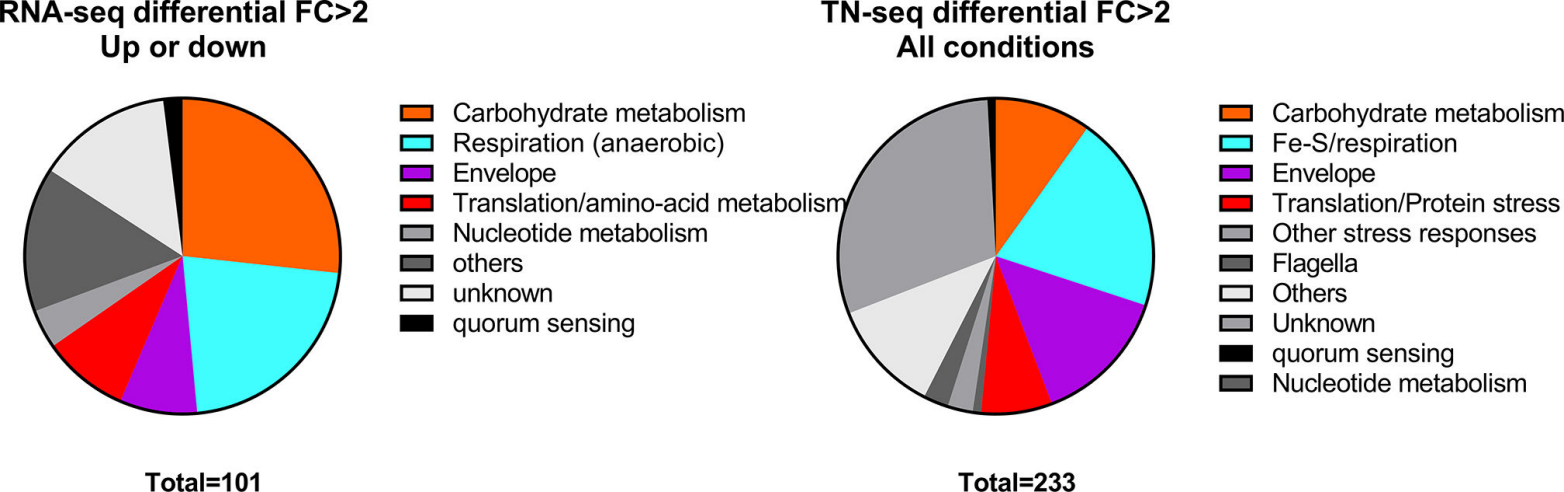
High-throughput methods identify factors that are differentially detected in *∆ravvia* compared to WT. Pie-chart for RNA-seq recapitulates differentially (up- or down-) expressed gene categories in *∆ravvia*, as shown in Table S1. Pie-chart for TN-seq recapitulates gene categories for differentially detected transposon insertions in *∆ravvia* compared to WT, in all conditions shown in Tables S2 to S4.

In order to further understand changes due to *ravvia* deletion and to search for potential partners of RavA-ViaA, we decided to adopt transcriptomic and TN-seq approaches (Fig. 3). RNA-seq was performed on exponentially growing WT and *∆ravvia*. Major changes in *∆ravvia* compared to WT include more than 10-fold upregulation of sugar transporters, anaerobic respiration, consistent with a recent study published by the Barras and Py laboratories (18) and (33), and the VC_1314-1315-1316 genes (Table S1).

The VC_1314 gene and the VC_1315-VC_1316 operon show 8- to 10-fold upregulation. VC_1315 presents 35% sequence identity which *E. coli zraS* gene. The ZraP regulator and ZraSR two-component membrane stress response system is involved in antibiotic resistance (25). VC_1315 also presents 29% (and 21%) sequence identity with the *V. cholerae* (and *E. coli) cpxA* gene. The periplasmic CpxP is the negative regulator of the envelope stress response CpxRA system (34). VC_1316 presents 26% sequence identity with the *E. coli cpxR* and 23% with the *V. cholerae cpxR*. VC_1314 does not show any sequence similarity neither to *zraP* nor to *cpxP*. In *E. coli*, the CpxP-CpxAR system and the ZraP-ZraRS systems were proposed to be functional homologs (26). We called VC_1314-1315-1316, the *zra-like* system below in this manuscript.

In parallel to the transcriptomic study, we applied our previously described comparative TN-seq approach (16, 19), to *V. cholerae ∆ravvia,* to search for genes that are important for survival in the presence of sub-MIC TOB, again in aerobic conditions. We sequenced mutant libraries before and after 16 generations without and with TOB at 50% of the MIC. After sequencing, comparative analysis of the number of detected gene inactivations between the two conditions indicates whether a given gene is important for growth in the antibiotic (decreased number of reads), or whether its inactivation is beneficial (increased number of raeds), or unchanged. Tables S2, S3, and S4 show the exhaustive lists of at least twofold differentially detected genes with transposon insertions in WT and *∆ravvia*, with and without TOB. Deletion of *ravvia* leads to changes in factors involved in carbon metabolism, iron and respiration, and membrane stress. We constructed deletion mutants in WT and ∆*ravvia* contexts and performed competition experiments (Fig. S2) for 21 of these genes to validate TN-seq results and to identify factors necessary for AG tolerance of *∆ravvia*. Competition results were mostly consistent with TN-seq data. Among identified factors, one, *cpxP*, has particularly caught our attention because its inactivation is beneficial in *∆ravvia* (Fig. S2), and because inactivation of *ravvia* in *∆cpxP* does not cause an additional increase in fitness, suggesting that they may act in the same pathway. Note that unlike CpxP, the *E. coli* ZraP represses the expression of the *zraPSR* operon only when bound to zinc (26). In the conditions of our TN-seq experiments in *V. cholerae*, there is no zinc supplementation, so if VC_1314 is a ZraP-like protein, it is not necessarily a repressor under the tested conditions. Upon envelope stress, *E. coli ∆zraP* shows increased membrane disruption (25). At T0, we counted 1.4-fold less insertions in *zraP-like* (VC_1314) in *∆ravvia*, than the WT, suggesting that in the absence of *ravvia*, ZraP function is more important. This is statistically significant (*p*adj = 7.3 × 10^−5^) but below the twofold change threshold and that’s why it does not appear in the TN-seq results tables. Because Cpx- and Zra-like systems were identified both in TN-seq and in RNA-seq, we decided to focus on the link between the AG tolerant phenotypes of *∆ravvia* and envelope stress response through the Cpx and Zra-like systems.

### Cpx and Zra-like system-mediated envelope stress response systems are necessary for the fitness advantage of *∆ravvia* during growth with sub-MIC AGs and AG tolerance

**Fig 4:**
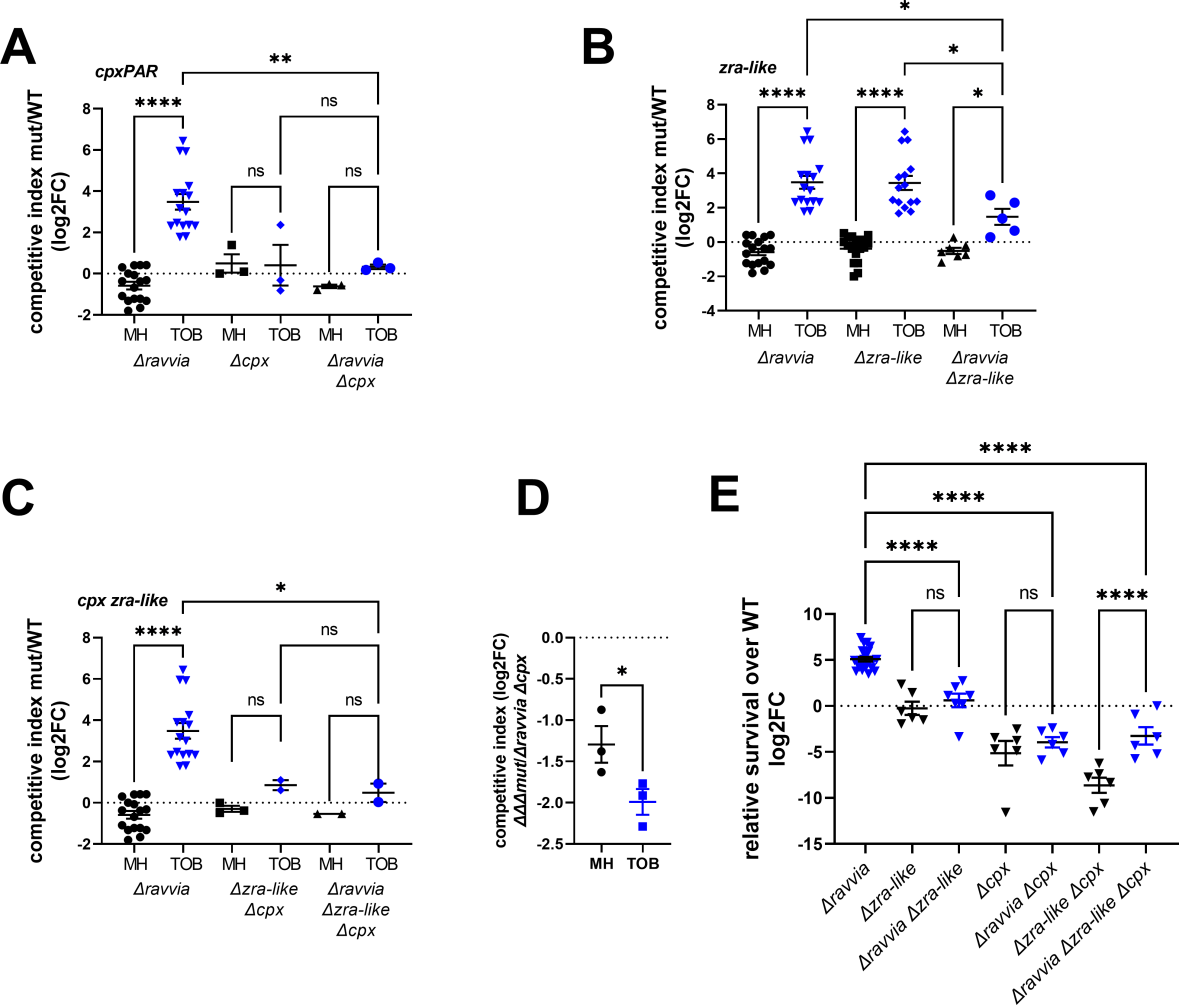
Cpx and Zra-like two-component envelope stress response systems are involved in fitness increase of *∆ravvia* with TOB and TOB tolerance. ABC. Competitions. The effect of deletion of *cpx* (**A**), or *zra-like* (**B**), or both (**C**) on competitive index in MH without and with TOB, where MH is the untreated growth medium. *In vitro* competition experiments of *V. cholerae* WT and indicated mutants in specified media: in black: MH: no antibiotic treatment. In blue: TOB: tobramycin 0.6 µg/mL. The *y*-axis represents log_2_ of competitive index value calculated as described in the methods. A competitive index of 1 (i.e., log_2_ value of 0) indicates equal growth of both strains. (**D**) Competitive index in MH without and with TOB, of the triple mutant (indicated as *∆∆∆*) *∆ravvia ∆cpx ∆zra* against *∆ravvia ∆cpx*. (E) Tolerance. Cultures were grown to exponential phase in MH medium. Survival of WT and *∆ravvia* to 3 h treatment with lethal TOB at 5× MIC 5 µg/mL was measured. The *y*-axis represents log_2_ value of survival ratios, calculated as survival of the mutant over survival of the WT. A relative survival ratio of 1 (i.e., log_2_ = 0) indicates equal survival as the WT strain. For statistical significance calculations, we used one-way ANOVA. **** means *P* < 0.0001, *** means *P* < 0.001, ** means *P* < 0.01, * means *P* < 0.05. ns, non-significant. Number of replicates for each experiment: 3 < *n* < 8.

In order to assess the importance of the Cpx and the putative Zra-like systems in the response to AGs of *∆ravvia,* we tested fitness and tolerance in competition and survival experiments in the absence of one or both of these systems. Fig. 4A through C shows competition experiments with *cpx* and *zra-like* operons inactivation (Fig. S3 shows the same competitions with statistical significance compared to the WT strain). Inactivation of *cpx* alone does not affect fitness in TOB (Fig. 4A), while inactivation of *zra-like* alone increases fitness in TOB (Fig. 4B). The fitness advantage of *∆zra-like* depends on the presence of *cpx* since deletion of *cpx* in *∆zra-like* suppresses its fitness advantage (Fig. 4C). Strikingly, the deletion of *cpx* or *zra-like* in *∆ravvia* leads to, respectively, loss or strong decrease of the fitness advantage of *∆ravvia* in TOB (Fig. 4A and B). The triple mutant *∆ravvia ∆cpx ∆zra* shows a phenotype similar to the *∆ravvia ∆cpx* double mutant (Fig. 4C). We performed additional competitions to ascertain the effect of deletion of *zra* in *∆ravvia ∆cpx*, this time by competing the triple *∆ravvia ∆cpx ∆zra* with *∆ravvia ∆cpx*. We observed that deletion of *zra* has, in fact, an additional negative impact on the fitness of *∆ravvia ∆cpx* (Fig. 4D). These results show that Cpx envelope stress response system is necessary for the enhanced tolerance of *∆ravvia* to TOB and that the Zra-like system also contributes significantly to this fitness advantage.

We next performed TOB tolerance tests using a concentration of 5× MIC for 3 h (Fig. 4E). Under these conditions, the survival of the single ∆*zra-like* system mutant is slightly lower than WT, the survival of *∆cpx* is lower, and both systems seem to be additive as the double mutant appears to show even lower tolerance than the single *∆cpx*. Strikingly, the high level of tolerance of *∆ravvia* is completely lost upon deletion of *zra-like* and goes even lower than WT upon deletion of *cpx* and in the triple mutant. For unknown reasons, the decrease of tolerance is stronger in *∆zra-like ∆cpx* than in *∆zra-like ∆cpx ∆ravvia*, as if deletion of *zra-like* in *∆ravvia ∆cpx* was beneficial. In any case, the AG tolerance conferred by the deletion of *ravvia* necessitates the presence of both Cpx and Zra-like systems.

### *∆ravvia-*related phenotypes are linked to extracellular zinc concentrations

**Fig 5:**
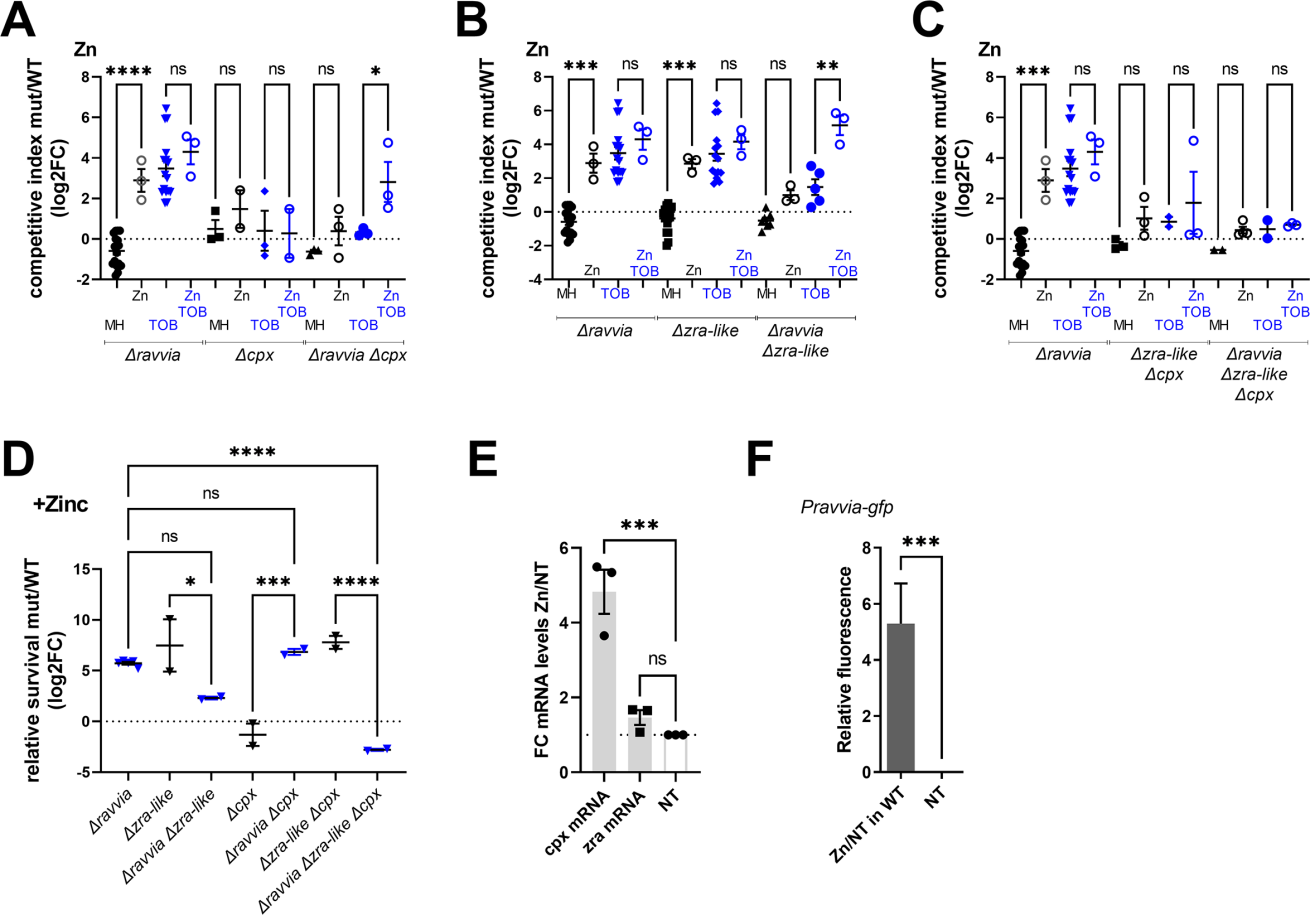
Effect of zinc supplementation. ABC. Competitions. Cultures were grown to exponential phase in MH medium supplemented with zinc during growth. “Zn” stands for ZnCl_2_: 1.5 mM. The effect of deletion of *cpx* (**A**), or *zra-like* (**B**), or both (**C**) on competitive index in MH without and with TOB, where MH is the untreated growth medium. *In vitro* competition experiments of *V. cholerae* WT and indicated mutants In specified media: in black: MH: no antibiotic treatment. In blue: TOB: tobramycin 0.6 µg/mL. The *y*-axis represents log_2_ of competitive index value calculated as described in the methods. A competitive index of 1 (i.e., log_2_ value of 0) indicates equal growth of both strains. (D) Survival of WT and *∆ravvia* to 3 h treatment with lethal TOB at 5× MIC 5 µg/mL, in the presence of zinc. The *y*-axis represents log_2_ value of survival rates ratios, calculated as survival of the mutant over survival of the WT. A relative survival ratio of 1 (i.e., log_2_ = 0) indicates equal survival as the WT strain. (E) Expression of *cpx* and *zra*. mRNA levels were measured using digital RT-PCR as explained in Materials and Methods. The *y*-axis represents the fold change of induction in the presence of zinc divided by the expression in the absence of zinc. (F) Expression from promoter of *ravvia* was measured using fluorescent transcriptional fusion of gfp expressed from *ravvia* promoter and quantified using flow cytometry. The *y*-axis represents the relative fluorescence in the indicated conditions. Zn: zinc 1.5 mM. NT: non-treated. ∆zur: zur deletion strain. WT: wild-type strain. For statistical significance calculations, we used one-way ANOVA. **** means *P* < 0.0001, *** means *P* < 0.001, ** means *P* < 0.01, * means *P* < 0.05. ns, non-significant. Number of replicates for each experiment: 3 < *n* < 6.

Since Cpx and Zra systems have previously been associated with metals such as iron and zinc, we next performed competition experiments in the presence of these metals. The presence of supplemented iron did not affect the fitness of the *∆ravvia* derivatives in any condition ( Fig. S4A through C). Zinc supplementation (Fig. 5Athrough C) restores fitness in TOB for the *∆ravvia ∆cpx* and *∆ravvia ∆cpx* double mutants but not for the triple mutant, suggesting that the effect of zinc in *∆ravvia* is somehow linked to the Cpx and Zra-like systems, which may act in a redundant way.

Moreover, while zinc has no effect on the TOB tolerance phenotype of *∆ravvia* or *∆ravvia ∆zra-like*, it restores high tolerance to the *∆ravvia ∆cpx* mutant (Fig. 5D), which is consistent with the zinc-dependent increase of fitness of the *∆ravvia ∆cpx* mutant shown in Fig. 5C. We wondered whether these genes could be regulated by zinc. We found that *cpx*, but not *zra*, mRNA levels are increased in the presence of zinc (Fig. 5E). Ravvia expression from P*ravvia* promoter fused to *gfp* is also induced by zinc (Fig. 5F). Overall, results indicate that the *∆ravvia* mutant’s AG tolerance is dependent on the Cpx and Zra-like systems and that *ravvia* function is also somehow associated with the sensing of zinc levels.

### *∆ravvia* has a low ROS phenotype which depends on Cpx/Zra-like systems

**Fig 6:**
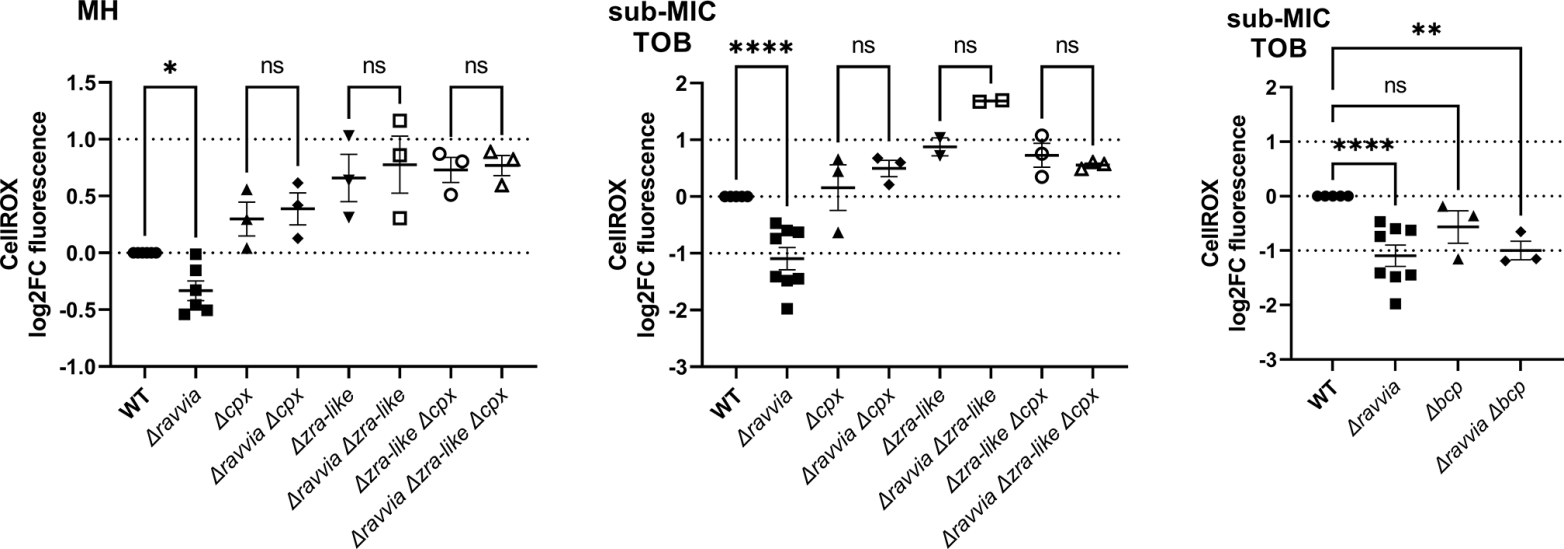
Low ROS phenotype of *∆ravvia* is dependent on the presence of Cpx and Zra-like stress response systems. Quantification of variation of reactive oxygen species using CellRox. The *y*-axis represents log_2_-fold change of detected ROS fluorescence in the indicated strain over the WT strain. Each experiment was performed at least three times, and data and statistical significance are shown in the histograms. For statistical significance calculations, we used one-way ANOVA. **** means *P* < 0.0001, ** means *P* < 0.01. ns means non-significant.

We have previously shown that sub-MIC TOB leads to reactive oxygen species (ROS) formation in *V. cholerae*, which induces the bacterial SOS stress response (35). However, *∆ravvia* is not more resistant to H_2_O_2_ (not shown). Considering that *∆ravvia* is more tolerant to TOB and that *∆ravvia* fails to induce SOS response in the presence of TOB (16), we hypothesized that these two observations could be explained by a diminished ROS formation in *∆ravvia*. We used the CellROX dye that, upon increased levels of ROS (O_2_^–^ and •OH), emits green fluorescence. We observed that lack of *ravvia* in *V. cholerae* leads to decreased ROS generation, both in the absence and in the presence of sub-MIC TOB (Fig. 6, MH and sub-MIC TOB). We similarly observed that *∆ravvia* produces decreased levels of ROS upon treatment with lethal doses of TOB (Fig. S5).

We next tested whether the function of Cpx/Zra-like systems in *∆ravvia* could be involved in the “low ROS” phenotype observed for *∆ravvia*. Fig. 6 shows that the deletions of either *cpx* or *zra-like* (or both) suppress this phenotype, meaning that both Zra-like and Cpx are involved in the low ROS phenotype of *∆ravvia*. This is consistent with the fact that both systems are also necessary for AG tolerant phenotype of *∆ravvia*. As a control, we also tested the double mutant *∆ravvia ∆bcp*. Bcp is a thiol peroxidase responding to oxidative stress. We see no effect of *bcp* deletion on the low ROS phenotype of *∆ravvia*. In conclusion, *∆ravvia* shows a low ROS phenotype, which is reversed upon inactivation of Cpx and Zra-like stress responses.

### Simultaneous deletion of Cpx or Zra-like systems with *∆ravvia* leads to outer membrane permeability

Since Cpx and Zra systems have been known for their involvement in envelope stress response, we decided to test the effect of *ravvia* deletion mutant and derivatives on outer membrane permeability.

**Fig 7:**
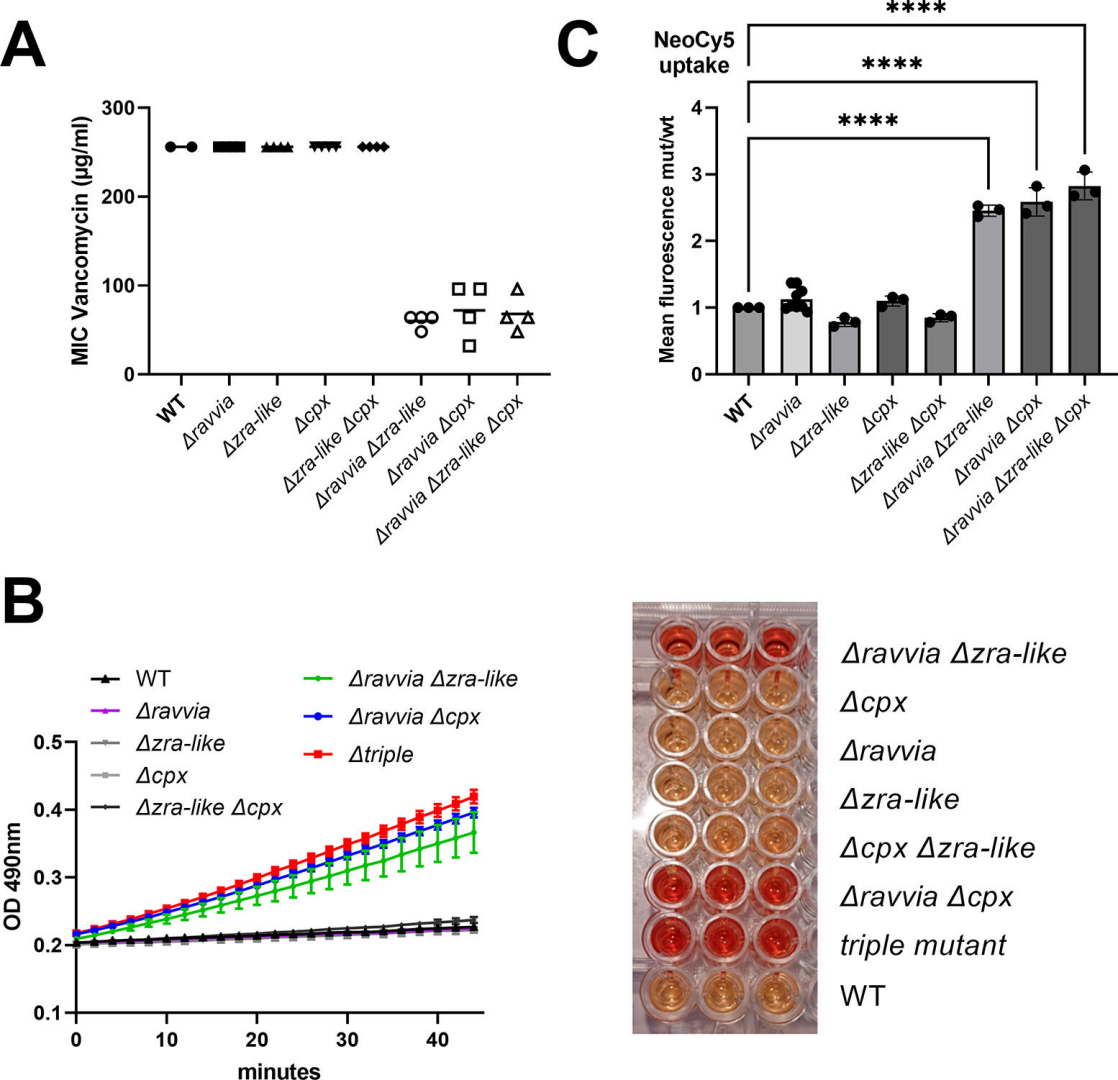
Response of *∆ravvia* to the membrane targeting antibiotics. (**A**) Minimum inhibitory concentration of vancomycin. MIC values are represented in the *y*-axis for the indicated strains. (**B**) Outer membrane permeability to nitrocefin measured on stationary phase cultures diluted to 5 × 10^7^ cells/mL by measuring the OD at 490 nm for 40 min. (**C**) AG uptake quantified through Neo-cy5 entry into exponential phase cultures. Intracellular level of neomycin coupled to the fluorophore Cy5 measured by fluorescence-associated flow cytometry. Error bars represent standard deviation. For statistical significance calculations, we used one-way ANOVA. **** means *P* < 0.0001. Only significant *P* values are shown.

Vancomycin is an antibiotic targeting the synthesis of the peptidoglycan, but which cannot be used to treat gram-negative bacteria, because its large molecular weight prevents it from crossing the outer membrane (OM) through porins, and penetrate into the cell (36). When the OM is damaged, however, vancomycin uptake by gram negative bacteria is possible (37, 38). We tested whether deletion of *ravvia* has an impact on vancomycin entry, by measuring the MICs of the different mutants. Our results show that single deletions of *∆ravvia*, *∆cpx,* or *∆zra-like* do not affect the MIC to vancomycin, while double deletions of *∆ravvia* together with *∆cpx* or *∆zra-like* or both decreases the MIC from >256 µg/mL to about 64–100 µg/mL (Fig. 7A), suggesting that simultaneous inactivation of *ravvia* function together with envelope stress response systems leads to OM damage or permeabilization.

Changes in OM permeability can be quantified using nitrocefin (39), a chromogenic probe which develops color (at 490 nm) upon entry into the periplasm, in the presence of β-lactamase. We, thus, measured permeability of *∆ravvia* and *∆cpx/∆zra-like* derivatives transformed with the low-copy pSC101 plasmid carrying the *bla* gene. Again, results show that neither single deletions of *∆ravvia*, *∆cpx,* or *∆zra-like* nor the double *∆cpx ∆zra-like* deletion affect nitrocefin entry, while double deletions of *∆ravvia* together with *∆cpx* or *∆zra-like* or both, strongly increases it, consistent with increased OM permeability (Fig. 7B).

Since AGs also are also expected to cross the cell envelope with higher efficacy with increased OM permeability, we checked whether AG uptake is increased in *∆ravvia ∆cpx* or *∆ravvia ∆zra-like* or the triple mutant (Fig. 7C). Consistent with vancomycin and nitrocefin entry results, neo-cy5 uptake is also increased in *∆ravvia ∆cpx, ∆ravvia ∆zra-like*, but not in single mutants or the *∆cpx ∆zra-like* mutant. Altogether, these results point to increased outer membrane permeability when either Cpx or Zra-like system is inactivated in *∆ravvia*.

## DISCUSSION

This study presents the first evidence of a link between RavA-ViaA function and the response to envelope stress. In fact, we identify the Cpx and a Zra-like two-component systems to be involved in the increased tolerance of *V. cholerae ∆ravvia* to AGs. Since Cpx and Zra systems are known to respond to protein misfolding at the periplasm, one can speculate that inactivation of *ravvia* could generate an increase in misfolded periplasmic proteins.

In *E. coli, ∆cpx* mutants show alterations in conjugational plasmid transfer, transport, ability to grow on some carbon sources, and resistance to AGs. More generally, the *E. coli* Cpx transcriptome impacts inner membrane-associated processes such protein secretion and other processes like iron homeostasis, translation, and, interestingly, ribosome protection factors *raiA* and *rmf* (34). Cpx also negatively regulates respiration, energy, and TCA cycle genes. In *V. cholerae*, Cpx senses and responds to low iron and induces iron transporters and efflux pumps (40). Disruption of the OM and accumulation of misfolded proteins in the periplasm were shown to induce the Cpx regulon (41 – 43). The fact that our TNseq data also identified the *tat* operon as important for the growth advantage of *∆ravvia* in TOB also supports the existence of a so far unknown link between RavA-ViaA and misfolded proteins in the periplasm. Cpx activity is regulated by successive phosphorylations (except for *cpxP*): envelope stress leads to phosphorylation and activation of CpxA, which phosphorylates and activates CpxR (44). Envelope stress in parallel inactivates the repressor CpxP because CpxP binds misfolded membrane proteins and is, thus, titrated away from CpxA. Thus, the Cpx activity is not solely regulated by activation of the promoter.

Such protein misfolding at the periplasm can happen during AG treatment, and Cpx was, in fact, previously linked to AG resistance (7, 45). Interestingly, Cpx was also shown to induce the heat-shock sigma factor RpoH in the presence of the AG gentamicin, indicating that the effect AG gentamicin at the membrane is sufficient to trigger the response to misfolded IM protein stress by Cpx (46). *∆cpxR* is more susceptible to AGs in *E. coli* (47), and in *Salmonella* independently of efflux pumps (48), and also independently of oxygen consumption or PMF (47), but involves altered protein composition at the membrane (49). The AG gentamicin was shown to activate the Cpx response (50). As a corollary, activation of Cpx leads to increased AG resistance. This was shown to be due to protection against AGs at the membrane (34, 51), partly through regulation of protein degradation at the inner membrane (49) and partly to downregulation of electron transport chains and iron import (34). Cpx was, in fact, described to repress *nuo* and *cyo* aerobic respiratory complexes in *E. coli* (47), which could be toxic in conditions challenging membrane integrity, and such repression allows bacterial cells to adapt to conditions disrupting membrane integrity, among which AG-induced protein misfolding. There are no described sequence homologs for *nuo* or *cyo* operons in *V. cholerae*. The functional equivalent may be the *nqr* system (52) (VC_2291-2-3-4), for which we see that inactivation is beneficial in WT TOB but not in *∆ravvia* TOB, maybe because it is already downregulated (approximately twofold but with *P*-value >0.05) in *∆ravvia*.

Since the Cpx response is involved in the biogenesis of large complexes present at the envelope (respiratory complexes, but also type 4 pili, and maybe others), one could envisage that the action of Cpx may be through reduced protein trafficking at the inner membrane, hence reduced membrane stress. These properties—decrease of respiration and iron import by Cpx—can explain how ROS formation increases in *∆ravvia* upon *cpx* deletion because ROS are mainly produced with oxygen and iron through the Fenton reaction. Cpx is also closely linked to energy status of the cell and regulates protein folding and degrading factors, which are involved in adaptation to stress caused by high level of respiration (53).

In *E. coli*, ZraPSR is involved in resistance to several drugs and is important for membrane integrity (25). Zra chaperone activity is enhanced by zinc. There is often significant overlap between processes affected by Zinc and by ROS, with zinc having mostly antioxidant function (54). *∆zra* has increased membrane disruption during treatment with membrane-targeting antibiotics. ChIP-seq and transcriptomic studies found that Zra controls the expression of *acr*, *raiA*, *rpoH*, etc. In *V. cholerae*, we find that the operon VC_1314-VC_1315-VC_1316 is highly upregulated in *∆ravvia*. Although VC_1314 (487 aa) does not present any sequence homology to neither *zra* nor *cpx* systems, VC_1315 (449 aa) and VC_1316 (158 aa) present homologies, respectively, with *E. coli zraS* (465 aa) and *cpxR* (232 aa). Since the *V. cholerae* VC_1314-VC_1315-VC_1316 *zra*-like system also responds to zinc, we propose to name it *zraP2-zraS2-zraR2*.

One question remains open: why does deletion of the RavA-ViaA function activate the Cpx/Zra2 systems? The answer could come from the fact that when both RavA-ViaA and Cpx or Zra2 are inactivated, membrane permeability increases as observed with vancomycin and nitrocefin entry. The mechanism could be a complex one since we also observe that RNA modifications, which impact translation (19), also impact *∆ravvia* phenotypes. Note that *V. cholerae* harbors two *groESL* operons, with *gro1* being essential and *gro2* accessory. We have recently shown the involvement of *gro2* in the response to AGs (28). Here, even in the absence of AGs, *gro2* is already essential for *∆ravvia*. A specific target of this chaperone may be important in the absence of *ravvia. ∆ravvia* increases resistance to AGs, but the importance of *gro2* and *clpS* may point to the existence of endogenous membrane protein stress in ∆*ravvia*. Thus, the presence of RavA-ViaA seems to be useful in order to maintain envelope integrity, and its function as a barrier against the entry of exogenous agents, such as the last resort antibiotic vancomycin.

## MATERIALS AND METHODS

Table S5 shows strains used in this study and their construction.

Table S6 shows primer sequences.

### Media and growth conditions

Platings were done at 37°C, in Mueller-Hinton (MH) agar media. Liquid cultures were grown at 37°C in MH in aerobic conditions, with 180 rotations per minute.

#### Statistical significance

For multiple comparisons, we used one-way ANOVA. **** means *P* < 0.0001, *** means *P* < 0.001, ** means *P* < 0.01, * means *P* < 0.05. ns, non-significant. All statistical tests were corrected for multiple comparisons and false discovery using the Bonferroni correction.

#### Competition experiments

Competition experiments were performed as described (19): overnight cultures from single colonies of mutant *lacZ*− and WT *lacZ*+ strains were mixed 1:1 (500 + 500 µL). At this point, 100 µL of the mix was serial diluted and plated on MH agar supplemented with X-gal (5-bromo-4-chloro-3-indolyl-β-d-galactopyranoside) at 40 µg/mL to assess t0 initial 1:1 ratio. At the same time, 5 µL from the mix was added to 200 µL of MH or MH supplemented with sub-MIC antibiotics (concentrations, unless indicated otherwise: TOB: tobramycin 0.6 µg/mL; GEN: 0.5 µg/mL; CIP: ciprofloxacin 0.01 µg/mL, CRB: carbenicillin 2.5 µg/mL), PQ: paraquat 10 µM, or H_2_O_2_: 2 mM. Cultures were incubated in 96 well plates with agitation at 37°C for 24 h and then diluted and plated on MH agar plates supplemented with X-gal. Plates were incubated overnight at 37°C, and the number of blue and white CFUs was assessed. Competitive index was calculated by dividing the number of white CFUs (*∆lacZ*− strain) by the number of blue CFUs (*lacZ*+ strain) and normalizing this ratio to the T0 (time zero) initial ratio.

### MIC determination

Stationary phase cultures grown in MH were diluted 20 times in PBS, and 300 µL was plated on MH plates and dried for 10 min. Etest straps (Biomérieux) were placed on the plates and incubated overnight at 37°C.

#### Survival/tolerance tests

Survival/tolerance tests were performed on early exponential phase cultures. The overnight stationary phase cultures were diluted 1,000× and grown until OD 600 nm of 0.35 to 0.4, at 37°C with shaking, in Erlenmeyers containing 25 mL fresh MH medium. Appropriate dilutions were plated on MH plates to determine the total number of CFUs in time zero untreated cultures. Five milliliters of cultures was collected into 50-mL Falcon tubes and treated with lethal doses of desired antibiotics (5 or 10 times the MIC: tobramycin 5 or 10 µg/mL, carbenicillin 50 µg/mL, ciprofloxacin 0.025 µg/mL) for 30 min, 1 h, 2 h, and 4 h if needed, at 37°C with shaking in order to guarantee oxygenation. Appropriate dilutions were then plated on MH agar without antibiotics, and proportion of growing CFUs was calculated by doing a ratio with total CFUs at time zero. Experiments were performed three to eight times.

#### Quantification of fluorescent neomycin uptake

Quantification of fluorescent neomycin uptake was performed as described (29). Neo-Cy5 is an aminoglycoside coupled to the fluorophore Cy5 and has been shown to be active against Gram-negative bacteria (31, 55). Briefly, overnight cultures were diluted 100-fold in rich MOPS (Teknova EZ-rich defined medium). When the bacterial strains reached an OD 600 nm of ∼0.25, they were incubated with 0.4 µM of Cy5-labeled neomycin for 15 min at 37°C. Ten microliters of the incubated culture was then used for flow cytometry, diluting them in 250 µL of PBS before reading fluorescence. WT *V. cholerae* was incubated simultaneously without Neo-Cy5 as a negative control. Flow cytometry experiments were performed as described (56) and repeated at least three times. For each experiment, 100,000 events were counted on the Miltenyi MACSquant device.

### PMF measurements

Quantification of PMF was performed using the Mitotracker Red CMXRos dye (Invitrogen) as described (32), in parallel with the neo-Cy5 uptake assay, using the same bacterial cultures. Fifty microliters of each culture was mixed with 60 µL of PBS. Tetrachlorosalicylanilide TCS (Thermofischer), a protonophore, was used as a negative control with a 500 µM treatment applied for 10 min at room temperature. Then, 25 nM of Mitotracker Red was added to each sample and let at room temperature for 15 min under aluminum foil. Twenty microliters of the treated culture was then used for flow cytometry, diluted in 200 µL of PBS before reading fluorescence.

### ROS measurements

Overnight cultures were diluted 1,000× in MH medium and grown until an OD 600 nm of 0.3. Then, 100 µL of each culture was transferred to a 96-well plate and treated with 1 µL of 250 µM CellRox Green (Thermofischer Scientific), for 30 min at 37 degrees, under aluminum foil. For flow cytometry, 10 µL was mixed into 200 µL of PBS. Fluorescence per cell was read on 100,000 events, on the MACSquant device at 488 nm.

### RNA purification and RNA-seq

Cultures were diluted 1,000× and grown in triplicate in MH to an OD 600 nm of 0.4. Frist, 1.5 mL of TriZol reagent was added to 500 µL of culture pellet followed by the addition of 300 µL of chloroforme. After centrifugation, the upper phase was mixed with a 1:1 vol of 70% ethanol before column purification. RNA was purified with the RNAeasy mini kit (Qiagen) according to manufacturer instruction (from step 4 of the protocole Part 1). Quality of RNA was controlled using the Bioanalyzer. Sample collection, total RNA extraction, library preparation, sequencing, and analysis were performed as previously described (57).

### Transposon insertion sequencing

Libraries were prepared as previously described (16, 58), to achieve a library size of 600,000 clones, and subjected to passaging in MH and MH + TOB 0.5 µg/mL for 16 generations (20). A saturated mariner mutant library was generated by conjugation of plasmid pSC1819 from *E. coli* to *V. cholerae* WT. Briefly, pSC189 (16, 58) was delivered from *E. coli* strain 7257 (β2163 pSC189::spec, laboratory collection) into the *V. cholerae* WT strain. Conjugation was performed for 2 h on 0.45 µM filters. The filter was resuspended in 2 mL of MH broth. Petri dishes containing 100 µg/mL spectinomycin were then spread. The colonies were scraped and resuspended in 2 mL of MH. When sufficient single mutants were obtained (>600,000 for 6× coverage of non-essential regions), a portion of the library was used for gDNA extraction using Qiagen DNeasy Blood & Tissue Kit as per manufacturer’s instructions. This was used for library validation through insert amplification by nested PCR using a degenerate primer (ARB6), which contains 20 defined nucleotides followed by a randomized sequence. This was combined with a primer anchored in the edge of the transposon sequence (MV288) (16, 20). After this, primer ARB3, which contains the first 20 nucleotides of ARB6, was used for nested amplification in combination with MV288. After validation, the libraries were passaged in MH media for 16 generations with or without 50% MIC of TOB, in triplicates. gDNA from time point 0 and both conditions after 16 generation passage in triplicate was extracted. Sequencing libraries were prepared using Agilent’s SureSelect XT2 Kit with custom RNA baits designed to hybridize the edges of the Mariner transposon. The 100 ng protocol was followed as per manufacturer’s instructions. A total of 12 cycles were used for library amplification. Agilent’s 2100 Bioanalzyer was used to verify the size of the pooled libraries and their concentration. HiSeq Paired-end Illumina sequencing technology was used producing 2 × 125 bp long reads. Reads were then filtered through transposon mapping to ensure the presence of an informative transposon/genome junction using a previously described mapping algorithm (59). Informative reads were extracted and mapped. Reads were counted when the junction was reported as mapped inside the CDS of a gene plus an additional 50 bp upstream and downstream. Expansion or decrease of fitness of mutants was calculated in fold changes with normalized insertion numbers. Normalization calculations were applied according to van Opijnen et al. (60). Expansion or decrease of fitness of mutants was calculated in fold changes with normalized insertion numbers. Baggerly’s test on proportions (61) was used to determine statistical significance as well as a Bonferroni correction for multiple hypotheses testing.

### mRNA quantifications by digital-RT-PCR

qRT-PCR reactions were prepared with 1 µL of diluted RNA samples using the qScript XLT 1-Step RT-qPCR ToughMix (Quanta Biosciences, Gaithersburg, MD, USA) within Sapphire chips. Digital PCR was conducted on a Naica Geode programmed to perform the sample partitioning step into droplets, followed by the thermal cycling program suggested in the user’s manual. Image acquisition was performed using the Naica Prism3 reader. Images were then analyzed using Crystal Reader software (total droplet enumeration and droplet quality control) and the Crystal Miner software (extracted fluorescence values for each droplet). Values were normalized against expression of the housekeeping gene *gyrA* as previously described (62).

### Quantification of *gfp* fusion expression by fluorescent flow cytometry

Flow cytometry experiments were performed as described (56) on overnight cultures and repeated at least three times. For each experiment, 50,000–100,000 events were counted on the Miltenyi MACSquant device.

### Transcriptional fusion

*ravvia* promoter sequence fused to *gfp* by amplification of *Pravvia-gfp* from pZE1-*gfp* (63) using primers ZIP537/ZIP200. The fragment was cloned into pTOPO-TA cloning vector. The *Pravvia-gfp* fragment was then extracted using *EcoRI* and cloned into the low copy plasmid pSC101. The plasmid was introduced into desired strains, and fluorescence was measured on indicated conditions, by counting 100,000 cells on the Miltenyi MACSquant device.

### Growth on microtiter plate reader

Overnight cultures were diluted 1:500 in fresh MH medium, on 96 well plates. Each well contained 200 µL. Plates were incubated with shaking on TECAN plate reader device at 37°C, and OD 600 nm was measured every 15 min. Tobramycin was used at sub-MIC: TOB 0.6 µg/mL. The concentrations of other antibiotics are specified in each figure.

### Quantification of nitrocefin entry

Nitrocefin, a chromogenic probe, 3-(2,4-dinitrostyryl)-(6 R,7 R)-7-(2-thienylacetamido)-ceph-3-em-4-carboxyl acid (Calbiochem), was used to assess membrane permeability. Tested strains were transformed with the low copy pSC101 plasmid carrying the *bla* gene coding for a β-lactamase, a periplasmic protein which allows coloration of nitrocefin upon entry into the bacterial cell. Cells were grown to stationary phase, washed twice, and resuspended at a concentration of 5 × 10^7^ cells/mL in PBS. The reaction was performed with 175 µL of PBS buffer and 25 µL of the nitrocefin 0.5 mg/mL stock solution, in 96 well plates. Fifty microliters of the bacterial suspension was added, and the OD at 490 nm was measured every 2 min for 45 min using a plate reader at 37°C, with shaking for 10 s every minute.

## Data Availability

The data discussed in this publication have been deposited in NCBI's Gene Expression Omnibus and are accessible through GEO Series accession number GSE196651 (GSM5897403, GSM5897404, GSM5897405, GSM5897412, GSM5897413, GSM5897414) for RNAseq data and GSE198341 for TNseq data (GSM5945317 , GSM5945318, GSM5945319, GSM5945323, GSM5945324, GSM5945325, GSM5945329, GSM5945330, GSM5945331, GSM5945338, GSM5945339, GSM5945340, GSM5945341, GSM5945342, GSM5945343).

## SUPPLEMENTAL MATERIAL

Supplemental figures spectrum.01730-23-s0001.pdf Fig. S1 to S5.

Supplemental tables spectrum.01730-23-s0002.pdf Tables S1 to S6.

ASM does not own the copyrights to Supplemental Material that may be linked to, or accessed through, an article. The authors have granted ASM a non-exclusive, world-wide license to publish the Supplemental Material files. Please contact the corresponding author directly for reuse.
