# Supplementary material for: RavA‐ViaA antibiotic response is linked to Cpx and Zra2 envelope stress systems in Vibrio cholerae
Source: Microbiol Spectr. 2023 Oct 20;11(6):e01730-23. doi: 10.1128/spectrum.01730-23 (PMC10848872; doi:10.1128/spectrum.01730-23)
Supplement: Supplemental figures — Fig. S1 to S5. [file spectrum.01730-23-s0001.pdf]

Supplementary figures

Figure S1

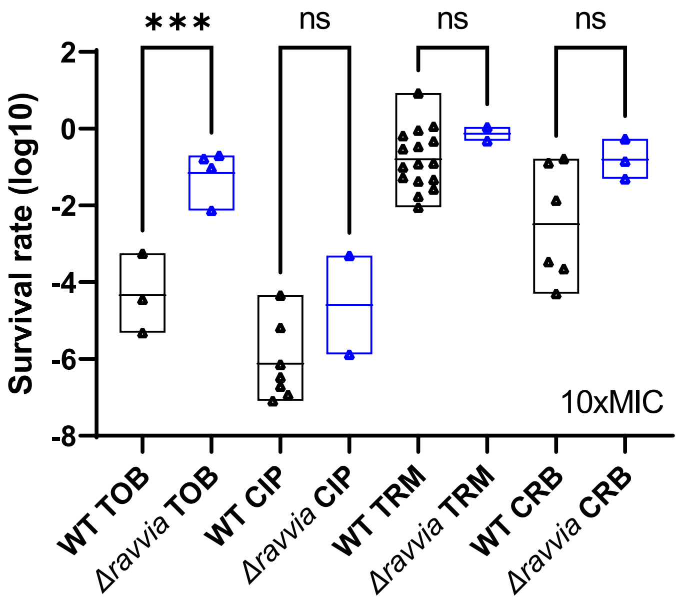

Figure S1: Effect of RavA-ViaA on tolerance to antibiotics. **A.** Survival of WT and *Δravvia* to treatment with antibiotics at 10x the MIC. Cultures were grown without antibiotics up to early exponential phase, and treated with antibiotics at lethal concentrations. TOB: tobramycin 10 µg/ml. Non-aminoglycoside antibiotics: CIP: ciprofloxacin. TRM: trimethoprim. CRB: carbenicillin. For statistical significance calculations, we used one-way ANOVA. *** means p<0.001, ns means non-significant. Number of replicates for each experiment: n>=3.

Figure S2

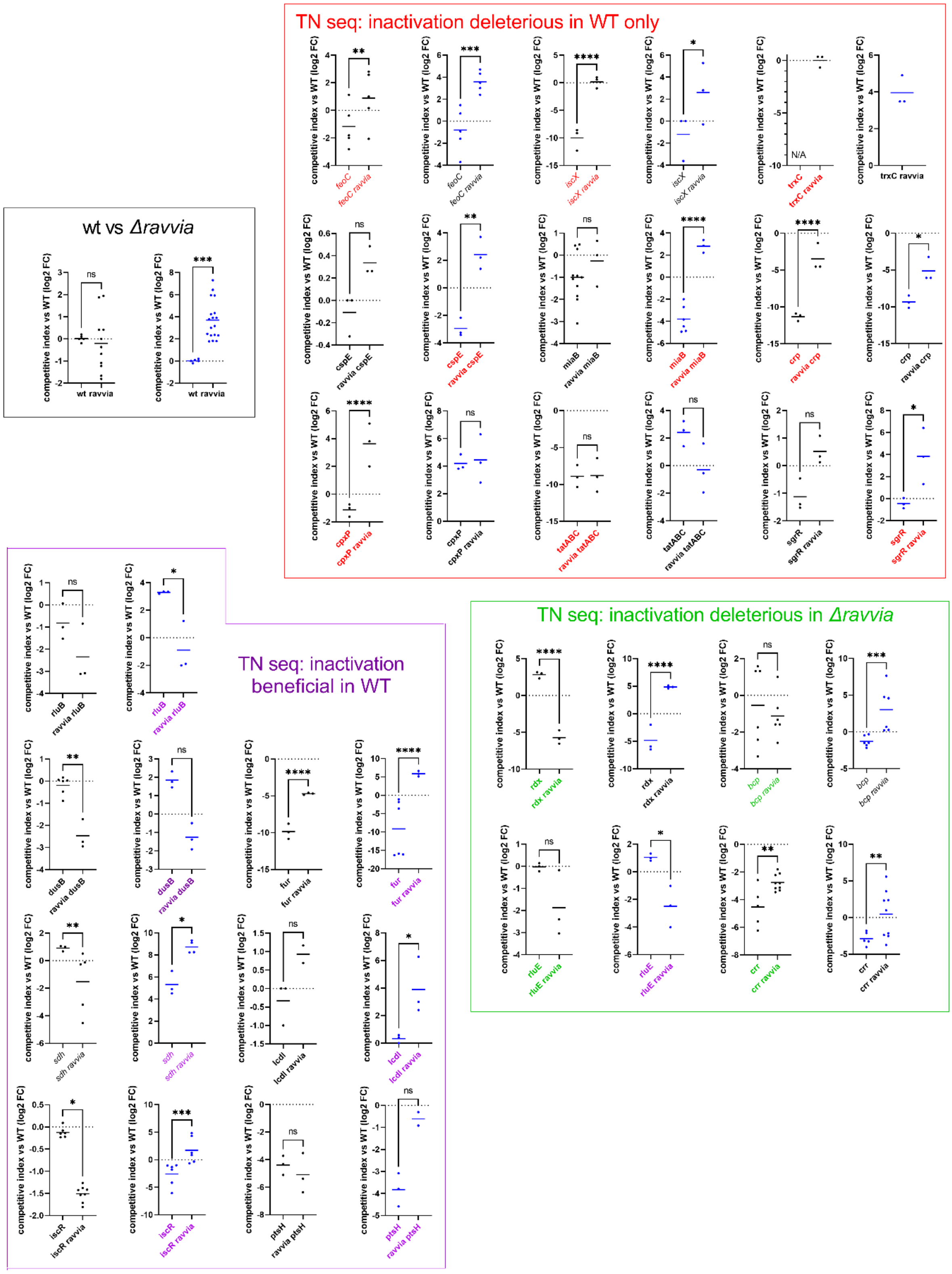
TN seq: inactivation deleterious in WT only
wt vs Δravvia
TN seq: inactivation beneficial in WT
TN seq: inactivation deleterious in Δravvia
competitive index vs WT (log2 FC)
N/A

Figure S2. Impact of selected gene deletions in WT and *Δravvia* on fitness during growth in sub-MIC antibiotics: *in vitro* competition experiments of *V. cholerae* WT and mutant strains in the absence or presence of TOB at sub-MICs (50% of the MIC). We were looking for factors for which inactivation would lead to loss of the fitness advantage of Δ*ravvia* in AGs. We tested the effect of 21 gene deletions, selected because they are important in WT but not in Δ*ravvia* (panel with red square), important in Δ*ravvia* (green panel) or beneficial in WT only (purple panel). For 16 of them, none completely suppressed this phenotype. TN-seq screen identified functions that are necessary for AG resistance in *Δravvia*. (i) in the absence of TOB: several genes are no longer essential or as important in *Δravvia* as in WT. These genes include carbohydrate utilization and metabolism genes (*crp*), iron and respiration related factors (*feoC, iscX, trxC*), and envelope related genes (*cpxP*). This suggests that deletion of *ravvia* leads to changes in carbon metabolism, iron and respiration, and membrane stress. This is consistent with transcriptomic data. Several genes become essential or important in *Δravvia:* genes involved in respiration and iron utilization (e.g. *rdx, bcp)*; carbohydrate metabolism (*crr*), translation (*truC, rluE*) and protein stress (*groES2, clpS,* not tested here in competition). (ii) after growth with TOB: several genes are no longer needed in *Δravvia:* electron transport/redox, carbohydrate metabolism (*sgrR*), stringent response and translation stress (e.g. *raiA, hpf,* not tested here), envelope and cell division, e*.g. cspE,* coding for a cold shock transcription anti-terminator which interacts specifically with mRNAs that encode membrane proteins [32]. In summary, several functions appear to be less needed in *Δravvia*: proteins protecting ribosomes upon translation stress (e.g. hibernation factors), consistent with the that AGs cause less translation stress in *Δravvia*. For cytochromes, their inactivation probably decreases PMF and confers AG resistance in WT but since *Δravvia* is already more resistant, their effect on PMF may have little impact on AG tolerance of *Δravvia*. Notable phenotypes were conferred by deletion of the *tat* operon (export of folded proteins to the perisplasm) which leads to loss of *Δravvia's* growth advantage in TOB, as well as RNA modification factors *dusB*, *rluB*, *rluE*, for which deletion is known to be beneficial in TOB [18], and cpxP which confers an advantage only to the WT strain. MH: no antibiotic treatment (black dots). TOB: tobramycin 0.6 µg/ml (blue dots). The Y-axis represents $\log_2$ of competitive index value calculated as described in the methods. A competitive index of 1 (i.e. log2 value of 0) indicates equal growth of both strains. Statistical comparisons are between the competition [*Δgene vs* WT] and [*Δgene Δravvia* vs WT]. For statistical significance calculations, we used one-way ANOVA. **** means p<0.0001, *** means p<0.001, ** means p<0.01, * means p<0.05. ns: non-significant. Number of replicates for each experiment: 3<n<6.

Figure S3

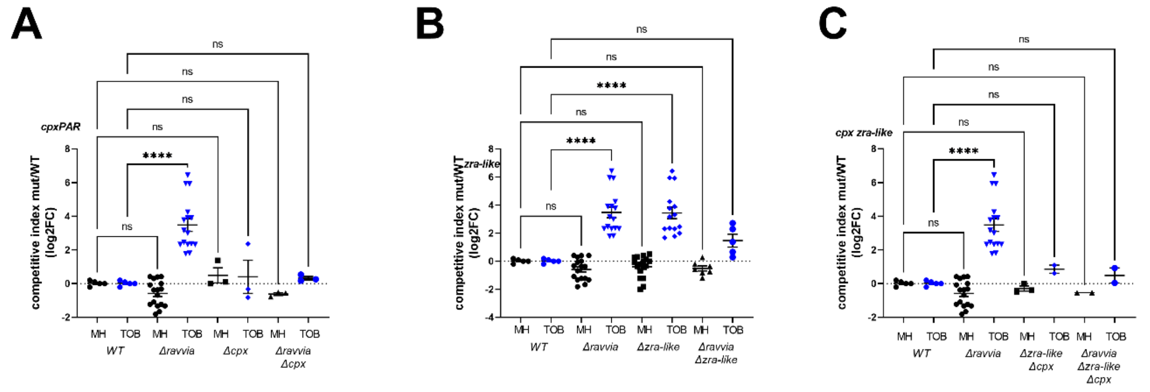

Figure S3. Cpx and Zra-like two component envelope stress response systems are involved in fitness increase of *Δravvia* with TOB and TOB tolerance. ABC. Competitions. The effect of deletion of *cpx* (**A**), or *zra-like* (**B**), or both (**C**) on competitive index in MH without and with TOB, where MH is the untreated growth medium. *In vitro* competition experiments of *V. cholerae* WT and indicated mutants in specified media: in black: MH: no antibiotic treatment. In blue: TOB: tobramycin 0,6 µg/ml. The Y-axis represents $\log_2$ of competitive index value calculated as described in the methods. A competitive index of 1 (i.e. log2 value of 0) indicates equal growth of both strains. Statistical significance compared to WT is shown. The data is the same as in Figure 4. **** means p<0.0001, *** means p<0.001, ** means p<0.01, * means p<0.05. ns: non-significant. Number of replicates for each experiment: 3<n<8.

Figure S4

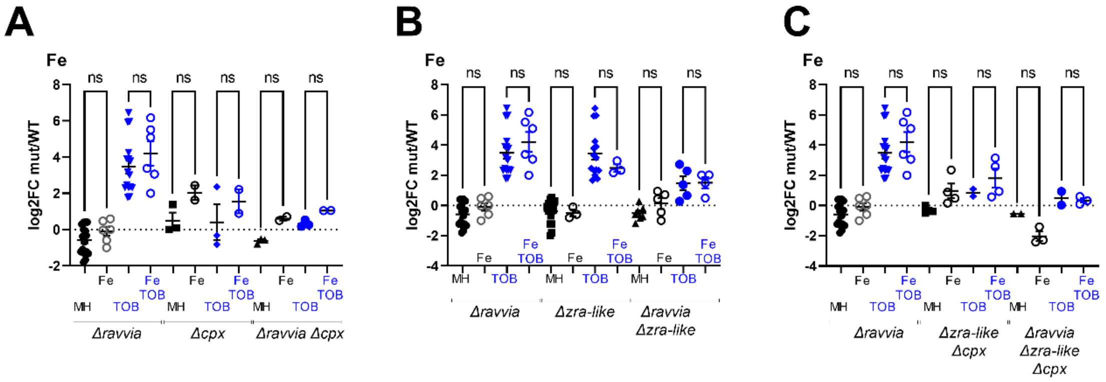

Figure S4: Effect of iron supplementation. ABC. Competitions. The effect of deletion of *cpx* (**A**), or *zra-like* (**B**), or both (**C**) on competitive index in MH with addition of iron, without and with TOB. Cultures were grown to exponential phase in MH medium supplemented with iron during growth. “Fe” stands for FeSO4 18µM. *In vitro* competition experiments of *V. cholerae* WT and indicated mutants in specified media: in black: MH: no antibiotic treatment. In blue: TOB: tobramycin 0,6 µg/ml. The Y-axis represents $\log_2$ of competitive index value calculated as described in the methods. A competitive index of 1 (i.e. log2 value of 0) indicates equal growth of both strains. For statistical significance calculations, we used one-way ANOVA. **** means p<0.0001, *** means p<0.001, ** means p<0.01, * means p<0.05. ns: non-significant. Number of replicates for each experiment: 3<n<8.

Figure S5

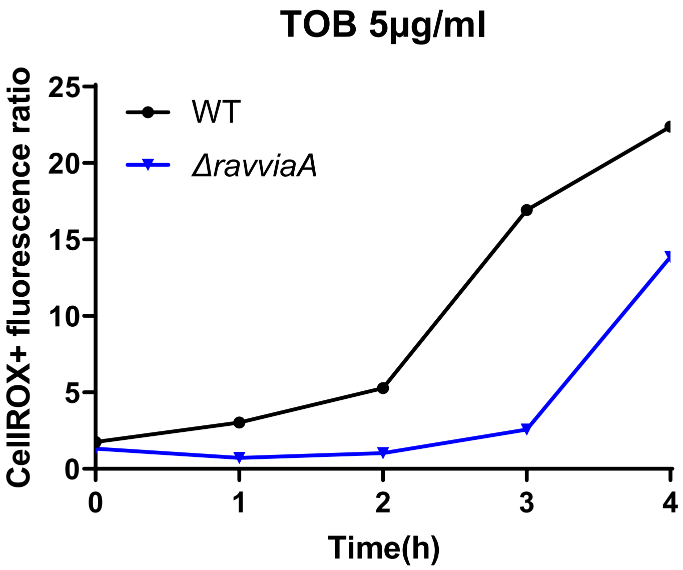

Figure S5: Low ROS phenotype of *Δravvia* is maintained in the presence of 5x MIC TOB. Quantification of variation of reactive oxygen species using CellRox. The y-axis represents fluorescence corresponding to detected ROS in the indicated strain, as a function of time. Experimental conditions were that of survival assays performed on exponentially growing cultures. Fluorescence was measured using flow cytometry every hour during TOB treatment, on 50,000 cells.
