# Supplementary material for: RavA‐ViaA antibiotic response is linked to Cpx and Zra2 envelope stress systems in Vibrio cholerae
Source: Microbiol Spectr. 2023 Oct 20;11(6):e01730-23. doi: 10.1128/spectrum.01730-23 (PMC10848872; doi:10.1128/spectrum.01730-23)
Supplement: Supplemental tables — Tables S1 to S6. [file spectrum.01730-23-s0002.pdf]

Table S1. RNA-seq

Locus tag	gene name	Fold change* increase in *Δravvia*
**Carbohydrate metabolism**		
**VC_1820**	putative PTS system, mannose specific IIA subunit	104.7
**VC_1821**	putative PTS system, maltose/mannose-specific IIBC component	11.5
**VC_1822**	putative PTS system mannose-specific IIA/IIB/IIC components	3.8
**VC_1825**	Putative ARAC-type regulatory mannose dependent	8.8
**VC_1826**	*manP*	27.4
**VC_1827**	*manA*	9.9
**VC_1325**	*mglB*	16.9
**VC_1327**	*mglA*	11.1
**VC_A0516**	*fruA*	10.4
**VC_A0517**	*pfkB*	10.7
**VC_A0518**	*fruB*	9.2
**VC_0687**	*cstA*	10.6
**VC_A0137**	*glpT*	14.0
**VC_A0747**	*glpA*	3.7
**VC_A0749**	*glpC*	3.4
**VC_1596**	*gal*	7.8
**VC_1595**	*galK*	9.8
**VC_1594**	*galM*	9.3
**VC_1898**	*trg*	4.7
**VC_1298**	*trg*	4.0
**VC_A1069**	*tar*	4.6
**VC_A0903**		3.2
**VC_2337**	*galR*	3.2
**VC_1645**		3.1
**VC_A0860**	*malS*	3.0
**VC_A1041**		2.5
**VC_A1045**		2.2
**Aminoacid/protein metabolism**		
**VC_1343**	*pepT*	11.5
**VC_0282**	*tsr*	4.4
**VC_0027**	*ilvA*	3.9
**VC_1872**	*yeaG*	2.7
**VC_A0574**	*patZ*	2.7
**VC_2374**	*gltD*	2.5
**VC_A0985**	*htpG*	3.3
**VC_A0885**	*tdh*	2.4

**VC_0392**		2.0
**Envelope**		
***VC_1315**	*zraS-like*	11.4
***VC_1316**	*zraR/cpxR-like*	10.9
**VC_1314**		8.8
**VC_A0867**	*ompW*	5.9
**VC_0972**	*chiP*	4.6
**VC_1081**	*ntrC*	3.1
**VC_1122**		2.2
**VC_1522**	*atoC*	2.1
**Respiration/redox**		
***VC_2656**	*frdA*	6.1
***VC_2657**	*frdB*	10.7
***VC_2658**	*frdC*	9.9
***VC_2659**	*frdD*	9.6
**VC_1950**	*torZ*	9.9
**VC_0651**	*ubiV*	6.7
**VC_1951**	*torY*	6.1
**VC_A0983**	*lldP*	6.0
**VC_A0984**	*lldD*	4.1
**VC_A0665**	*dcuC*	5.4
**VC_A0205**	*dcuB*	5.1
**VC_0338**		5.1
**VC_A0784**		4.6
**VC_A0610**	*elbB*	4.3
**VC_A0691**		4.2
**VC_A0512**	*nrdG*	3.6
**VC_A0690**		3.5
**VC_1514**	*tatB*	2.9
**VC_1512**	*ynfG*	2.8
**VC_1515**	*torD*	2.5
**VC_1511**		2.4
**VC_1516**		2.3
**Nucleotide metabolism**		
**VC_A0053**		3.3
**VC_A0592**		3.1
**VC_A0798**	*yieH*	2.9
**VC_1034**	*udp*	2.6
**Others**		
**VC_2361**	*grcA*	6.6
**VC_0076**	*uspA*	4.1
**VC_A0904**	*gntP*	3.5
**VC_A1015**		3.3
**VC_0737**	*acuB*	2.9
**VC_1605a**		2.9

**VC_A0689**		2.7
**VC_0728**	*ppk2*	2.7
**VC_A0221**		2.6
**VC_A0274**		2.5
**VC_A0688**	*phaC*	2.4
**VC_0551**		2.3
**VC_0550**	*oadA*	2.3
**VC_2507**	*ybeZ*	2.1
**VC_2473**	ytfK	3.8
**Quorum Sensing, Competence**		
**VC_A0865**	*hapA*	2.4
**VC_1153**	*tfoX*	4.2
**Hypotetical proteins**		
**VC_A1065**		4.7
**VC_1080**		3.9
**VC_0957**	*ybeL*	3.6
**VC_A0619**		3.0
**VC_1690**		2.8
**VC_2470**		2.5
**VC_1710**		2.2
**VC_1698**		2.2
**VC_A0971**		2.1
**VC_A0330**		2.1
**VC_1125**		2.1
**VC_A0381**		2.1
**VC_1697**		2.0
**VC_2221**		12.8
**Locus tag**	**gene name**	**Fold change* decrease in *Δravvia***
**Controls**		
**VC_A0763**	*ravA*	250
**VC_A0762**	*viaA*	142.9
**Purine biosynthesis**		
**VC_2227**	*purN*	3.6
**VC_2226**	*purM*	3.4
**VC_1004**	*purF*	2.5
**Translation, tRNA synthesis**		
**VC_2706**	*yhhQ*	3.5
**VC_1942**	*folD*	2.8
**VC_0327**	*rplL*	2.2
**VC_0564**	*rplS*	1.9
**VC_0325**	*rplA*	1.9
**VC_1259**	*trhO*	2.7
**VC_t017**	*tRNA-Thr*	2.1
**VC_A1059**	*trmY*	2
**VC_0916**	*etp*	2.7

**Aminoacid transport and synthesis**		
**VC_0907**	*metN*	2.1
**VC_1312**	*alr*	2.1
**VC_1658**	*sdaC*	3.3
**VC_A0063**	*ptrB*	3.1
**Iron-sulfur and respiration**		
**VC_0538**	*cysP*	2.5
**VC_0384**	*cysJ*	2.4
**VC_A0064**	*tonB*	2.3
**VC_0749**	*iscU*	2
**VC_1623**	*nspC*	2.1
**VC_1624**		2.4
**VC_A0779**	*puuB*	2.4
**Others**		
**VC_2561**	*cobA*	2.8
**VC_1040**	*cobO*	2.3
**VC_1112**	*bioB*	2.6
**VC_A0035**		2.1

***: only values with adjusted pvalue<0.01 are shown**

Table S2. Transposon insertion sequencing at time T0: genes that cannot be inactivated only in WT or only in *Δravvia*

Non-treated – No insertion detected in WT (no reads) but inactivation possible in Δravvia	
**Carbohydrate metabolism**	
csrA	Carbon storage regulator, stringent response
bax (VC_1430)	Sugar metabolism (glycoside hydrolase)
crp*	Carbon catabolite control (enriched in ravvia but still very low)
**Iron**	
yggX (VC_0451)	Role in Fe-S cluster stability, redox sensitive
feoC*(VC_2076)	Ferrous iron uptake, redox sensitive FE-S cluster, Fur regulation
iscX*	Regulator of Fe-S assembly, function unclear
**Redox/respiration**	
trxC*	Thioredoxin, redox sensitive
dsbD (VC_A0325)	Respiration, thiol:disulfide interchange protein
ubiG (VC_1262)	Respiration, 3-demethylubiquinone-9 3-methyltransfera
**Envelope**	
cpxP*(VC_2691)	Resistance to extracytoplasmic stress, envelope stress (with degP)
rffM (VC_0927)	Enterobacterial common antigen
tatB* (	Export of folded proteins
**Others**	
ligA VC_1542)	DNA ligase
gspS2 (	Lipoprotein
higB-1 (VC_A0391)	Toxin
**Unknown**	
yggU	VC_0458
yhbS	VC_0655
**hyp**	VC_1613
**hyp**	VC_1637
**hyp**	VC_A0381
**pseudogene**	VC_A0390
**hyp**	VC_A0467
**hyp**	VC_A0471
**hyp**	VC_A0547
**hyp**	VC_A1030
**hyp**	VC_0868
**Non-treated – No insertion detected in *Δravvia* (no reads) but inactivation possible in WT**	
**Expected control genes**	
lacZ	No reads in *Δravvia* because the strain is *ΔlacZ*
viaA	
ravA	
**Redox/respiration/iron**	
napC VC_A0680	Cytochrome c
doxX VC_A1019	Oxydoreductase
rdx* VC_0982	Oxidoreductase (selenoprotein W-related protein/selT motif)

grx4 **VC_2044**	Glutaredoxin
bcp* **VC_2160**	Thioredoxin-Dependent Thiol Peroxidase (homol. to selU YbbB tRNA 2-selenouridine synthase)
**VC_0382**	Putative ABC-type Fe3+-hydroxamate transport system
**Carbohydrate metabolism**	
crr*	Phosphotransfer protein in sugar uptake, regulates sugar metabolism
ace	Isocitrate lyase (deletion =>carbon catabolite repression)
creA* VC_A0800	Catabolite regulation protein
**Translation/protein stress**	
rrf_9 VC_r022	5S Ribosomal RNA, polar on tRNA glu/lys/val
epmC VC_2113	efp hydroxylase
truC	tRNA pseudouridine65 synthase (ile, asp)
rluE*	23S rRNA pseudouridine2457 synthase, P center of the ribosome, associated to AG resistance
clpS	protease
groES2 VC_A0819	chaperone
**Stress**	
crl	stat phase/stress regulator (rpoS regulon)
VC1615	UTP pyrophosphatase, prevent unspecific incorporation of modified bases into RNAs
ytfK VC_2473	Stringent response
**Others**	
flgJ	peptidoglycan hydrolase , creates holes in the peptidoglycan layer for flagella assembly
VC_0246	LPS transport system permease protein
trpR	tryptophan (trp) transcriptional repressor
**Unknown:**	
**VC_0124**	putative lipoprotein L
**VC_1176**	Trp operon leader peptide TrpL
**VC_1537**	putative Lipoprotein NlpC
**VC_2040**	hypothetical
**VC_2147**	hypothetical
**VC_A0233**	hypothetical
**VC_A0348**	Toxin RelE
**VC_A0435**	hypothetical
**VC_A0497**	Toxin DhiT
**VC_A0652**	hypothetical
**VC_A0831**	hypothetical
**VC_A0966**	hypothetical
**VC_A1061**	hypothetical

Table S3. Transposon insertion sequencing at time T16, with no treatment: genes that tolerate more insertions either in WT or in *Δravvia*

**Non- treated 16 gen– More insertions in ravvia**										
**gene name**		**T0 ravvia**	**T16 ravvia**	**T16 TOB ravvia**	**T0 WT**	**T16 WT**	**T16 TOB WT**	**FC T0 ravvia/wt**	**FC T16 * ravvia/wt**	**FC T16 TOB ravvia/wt**
**carbohydrate metabolism**										
araD	VC_A0244	194	174	143	58	110	97	3.3	**1.6**	**1.5**
malK		510	242	352	140	96	142	3.6	**2.5**	**2.5**
	VC_0549	482	232	334	152	222	173	3.2	**1.0**	**1.9**
**redox**										
	VC_A0506	234	88	212	69	165	108	3.4	**0.5**	**2.0**
**nucleotide pool**										
surE/umpG		409	352	323	140	152	69	2.9	**2.3**	**4.7**
pnpP	VC_A0970	331	212	204	91	241	132	3.6	**0.9**	**1.5**
**envelope/membrane**										
	VC_A0152	410	422	344	112	320	150	3.7	**1.3**	**2.3**
mshF	VC_0407	869	525	618	154	127	151	5.6	**4.1**	**4.1**
vpsQ	VC_0939	901	679	783	292	313	283	3.1	**2.2**	**2.8**
**others**										
mobA		212	141	151	63	67	71	3.4	**2.1**	**2.1**
citX		411	331	311	117	203	86	3.5	**1.6**	**3.6**
trpD		149	142	187	63	60	59	2.4	**2.3**	**3.2**
phhB	VC_A0827	546	391	384	154	178	148	3.5	**2.2**	**2.6**
**unknown**										
alpA	VC_1809	286	373	383	90	112	183	3.2	**3.3**	**2.1**
yebG	VC_2326	340	237	286	107	156	110	3.2	**1.5**	**2.6**
ynjD	VC_1666	206	225	222	32	98	101	6.3	**2.3**	**2.2**
hyp	VC_A0743	259	241	258	50	81	67	5.2	**3.0**	**3.9**
hyp	VC_A0440	353	355	421	78	139	69	4.5	**2.6**	**6.1**
yaeP	VC_0872	395	348	271	91	184	201	4.3	**1.9**	**1.3**
**hyp**	VC_A0086	297	271	267	71	254	191	4.2	**1.1**	**1.4**
**hyp**	VC_A0649	360	331	318	93	188	195	3.9	**1.8**	**1.6**
**hyp**	VC_A0032	516	417	542	137	158	114	3.8	**2.6**	**4.8**
**?**	VC_A0429-30	195	262	375	52	113	149	3.7	**2.3**	**2.5**
**hyp**	VC_A1030	173	137	115	55	40	58	3.1	**3.5**	**2.0**
yecM	VC_2073	191	205	181	61	134	120	3.1	**1.5**	**1.5**
**Non treated 16 gen– more insertions in WT**										
**gene name**		**T0 ravvia**	**T16 ravvia**	**T16 TOB ravvia**	**T0 WT**	**T16 WT**	**T16 TOB WT**	**FC T0 wt/ravvia**	**FC T16* wt/ravvia**	**FC T16 TOB wt/ravvia**
**Translation/protein**										
yjgA	VC_2536	51	96	77	163	172	142	3.2	**1.8**	**1.8**
**membrane transport**										
	VC_1605a	16	31	38	198	146	99	12.5	**4.7**	**2.6**

pspG		56	194	113	561	579	421	10.0	**3.0**	**3.7**
		38	6	25	148	22	27	3.9	**3.4**	**1.1**
yhdZ	VC_A1037	55	93	128	177	165	165	3.2	**1.8**	**1.3**
**carbohydrate metabolism**										
pfkA		31	13	28	124	54	62	4.0	**4.0**	**2.2**
galU		68	48	23	215	100	16	3.2	**2.1**	**0.7**
**regulators**										
cspA	VC_A0166	30	24	28	115	162	32	3.8	**6.7**	**1.1**
marR	VC_A1005	37	92	65	181	190	110	4.8	**2.1**	**1.7**
	VC_A0999	65	65	69	239	144	123	3.7	**2.2**	**1.8**
**FE-S/redox**										
dsbD	VC_A0389	39	99	101	155	195	103	4.0	**2.0**	**1.0**
ibaG	VC_2515	8	19	9	114	91	6	14.3	**4.8**	**0.7**
grxB		36	43	47	129	119	68	3.6	**2.7**	**1.5**
	VC_1254	61	102	190	216	193	387	3.5	**1.9**	**2.0**
hutC		55	72	88	187	164	127	3.4	**2.3**	**1.4**
speB		26	41	19	109	73	35	4.2	**1.8**	**1.9**
thiS		63	38	78	202	157	105	3.2	**4.1**	**1.3**
**electron transfer**										
ccmF	VC_A0368	88	136	103	311	224	201	3.5	**1.6**	**2.0**
ccmE		61	36	81	190	108	846	3.1	**3.0**	**10.4**
cobO		73	77	82	221	180	165	3.0	**2.3**	**2.0**
**DNA repair**										
xthA		37	38	37	140	158	141	3.8	**4.1**	**3.8**
**two component system**										
dpiB citA*	VC_0791	48	67	65	179	161	119	3.7	**2.4**	**1.8**
cheY	VC_A1096	61	39	62	207	154	36	3.4	**4.0**	**0.6**
**others**										
hfq		33	38	0	117	116	0	3.5	**3.1**	**no reads**
**unknown**										
**hyp**	VC_2367	22	103	85	199	173	160	9.0	**1.7**	**1.9**
yacL	VC_0605	59	125	131	437	371	224	7.5	**3.0**	**1.7**
**hyp**	VC_A0494	24	35	14	113	141	139	4.8	**4.0**	**10.2**
**hyp**	VC_A0919	60	107	68	284	174	260	4.7	**1.6**	**3.8**
ybhG	VC_1659	33	78	73	143	154	104	4.3	**2.0**	**1.4**
**hyp**	VC_A0458	92	127	126	369	275	183	4.0	**2.2**	**1.5**
fliT		75	390	89	296	869	23	4.0	**2.2**	**0.3**
**hyp**	VC_2046	143	202	153	539	562	281	3.8	**2.8**	**1.8**
**hyp**	VC_A0387	145	172	183	545	363	288	3.7	**2.1**	**1.6**
rstA2	VC_1463	68	17	0	253	205	138	3.7	**11.7**	**no reads in ravvia**
wbeT rfbT	VC_0258	37	46	36	136	154	93	3.7	**3.3**	**2.5**
unfA	VC_1191	48	81	125	176	128	78	3.6	**1.6**	**0.6**
**hyp**	VC_A1024	136	227	217	483	597	369	3.6	**2.6**	**1.7**
**hyp**	VC_0491	39	81	68	138	123	90	3.5	**1.5**	**1.3**

**hyp**	VC_A0342	59	112	125	189	133	113	3.2	**1.2**	**0.9**

***: FC T16, only values with adjusted pvalue<0.01 are shown**

Table S4. Transposon insertion sequencing at time T16, with sub-MIC TOB: genes that tolerate more insertions either in WT or in *Δravvia*

gene name	T0 ravvia	T16 ravvia	T16 TOB ravvia	T0 WT	T16 WT	T16 TOB WT	fold decrease ravvia T16TOB/T0	fold decrease WT T16TOB/T0	FC T16 TOB ravvia/wt **	
**TOB 16gen: insertions decrease in wt but unchanged (or increased) in ravvia => factors needed in TOB in WT but not needed in Δravvia**										
**electron transport/redox**										
**VC_A0186**	22	92	**138**	44	60	**21**	0.7	2.9	**6.7**	NADH:ubiquinone oxidoreductase subunit 2
**VC_A0463**	76	177	**128**	39	89	**39**	1.4	2.3	**3.3**	glyoxalase
ahpC	219	221	**174**	179	107	**17**	1.3	6.2	**10.1**	**peroxiredoxin C**
ypjD	289	300	**246**	243	203	**71**	1.2	2.9	**3.5**	cytochrome c assembly ypjD
ridA	217	145	**154**	267	191	**21**	0.9	9	**7.2**	**RidA family redox-regulated chaperone**
ubiG	120	153	**146**	187	172	**41**	1	4.2	**3.5**	
potD	470	425	**314**	249	266	**82**	1.4	3.2	**3.8**	spermidine preferential ABC transporter
dksA	117	299	**233**	111	310	**31**	1.3	10	**7.5**	
thiD	200	178	**217**	89	136	**64**	0.8	2.1	**3.4**	
trpB	164	137	**131**	94	84	**18**	1	4.5	**7.1**	
	**Translation**									
raiA	103	101	**156**	124	52	**17**	0.6	3.1	**9.2**	ribosome protection
yeiP	214	208	**211**	277	271	**50**	1	5.4	**4.2**	EFP like protein
hpf	261	170	**114**	155	81	**22**	1.5	3.6	**5.1**	ribosome hibernation
sgrR	212	224	**225**	119	147	**56**	1	2.6	**4**	Sugar transport-related Regulator
**envelope/membrane/cell division**										
ompR	278	211	**167**	198	174	**40**	1.3	4.4	**4.2**	
envC	357	349	**294**	271	316	**92**	1.2	3.4	**3.2**	murein hydrolase activator (septum)
envZ	393	340	**298**	310	258	**71**	1.1	3.6	**4.2**	
envZ	275	289	**161**	175	235	**47**	1.8	5	**3.4**	sensory histidine kinase
zapD	294	286	**282**	195	197	**75**	1	2.6	**3.8**	
matP	110	215	**183**	131	139	**14**	1.2	10	**13.2**	
ftsE	464	476	**331**	388	421	**63**	1.4	6.7	**5.3**	
ftsX	493	477	**447**	427	470	**77**	1.1	6.1	**5.8**	
DedD	146	298	**178**	76	173	**15**	1.7	11.7	**12**	cell division protein
RlpA	313	335	**216**	313	306	**47**	1.6	6.5	**4.6**	septal ring lytic transglycosylase
YfbV	302	240	**210**	268	303	**30**	1.1	9.9	**6.9**	membrane protein
Blc	372	424	**384**	195	286	**94**	1.1	3	**4.1**	OM lipoprotein
glpG	243	227	**176**	333	323	**33**	1.3	9.8	**5.3**	serine proteases which cleave membrane proteins

cspE*		164	261	**135**	60	118	**1**	1.9	185.2	**211**	cold shock transcription antiterminator interact specifically with mRNAs that encode membrane proteins
nhaB		380	356	**279**	203	224	**76**	1.3	2.9	**3.7**	Na+:H+ antiporter
others											
hapR		397	645	**381**	295	477	**54**	1.7	8.9	**7.1**	**Quorum sensing master regulator**
menC		376	328	**341**	178	136	**71**	1	1.9	**4.8**	menaquinone synth. (vitK2)
bioD		234	180	**135**	160	194	**37**	1.3	5.3	**3.7**	biotin synth
fklB		324	379	**117**	244	165	**27**	1	4.2	**3.2**	peptidyl-prolyl cis-trans isomerase
diaA		173	193	**202**	138	110	**59**	1	1.9	**3.4**	DnaA initiator-associating factor for replication initiation
unknown											
***yfcL* VC_2112**		83	126	**236**	60	43	**51**	0,4	121.3	**4.7**	
**hyp**	VC_1810	80	134	**156**	55	109	**33**	0.5	213.8	**4.7**	
**hyp**	VC_A0003	112	129	**203**	41	114	**60**	0.6	206.7	**3.4**	
**hyp**	VC_A0302	183	241	**281**	190	155	**50**	0.7	237.9	**5.6**	
**hyp**	VC_1531	160	138	**244**	62	190	**65**	0.7	290.4	**3.7**	
tldD		262	334	**358**	210	272	**87**	0.7	372.0	**4.1**	
plsY		167	144	**116**	251	173	**36**	1.4	120.5	**3.2**	
yhbS	VC_0655	329	261	**275**	139	78	**32**	1.2	65.8	**8.5**	
	VC_A0808	118	230	**121**	212	270	**6**	1.0	277.6	**21.2**	
phnX	VC_A0606	204	238	**262**	106	108	**86**	0.8	138.4	**3.1**	
hyp	VC_1613	280	357	**352**	121	111	**75**	0.8	140.0	**4.7**	
YeeX	VC_A0741	168	171	**177**	84	138	**2**	0.9	145.7	**74.7**	
**hyp**	VC_1262	143	298	**171**	77	63	**23**	0.8	75.5	**7.4**	
**hyp**	VC_1479	313	323	**303**	171	229	**90**	1.0	221.3	**3.4**	
**hyp**	VC_A0631	121	167	**168**	74	86	**43**	0.7	119.6	**3.9**	
**hyp**	VC_2434	347	294	**297**	216	176	**97**	1.2	150.5	**3.1**	
**hyp**	VC_1574	213	219	**286**	156	151	**93**	0.7	202.4	**3.1**	
**hyp**	VC_1310	192	149	**185**	144	151	**49**	1.0	146.2	**3.8**	
**hyp**	VC_A0382	262	232	**251**	220	146	**75**	1.0	140.7	**3.4**	
	VC_1536	129	172	**192**	131	122	**52**	0.7	182.4	**3.7**	
	VC_A0331-2	103	150	**119**	112	126	**22**	0.9	146.1	**5.5**	
**TOB 16gen: insertions highly decrease in wt but slightly decreased in ravvia => factors needed in TOB in WT but less needed in Δravvia**											
**translation**											
miaB*		251	234	**119**	253	233	**39**	2	6	**3.1**	tRNA modification
rsmI		378	299	**122**	441	297	**34**	2.4	8.6	**3.6**	rRNA modification
**flagella**											
fliR		153	663	**314**	118	684	**73**	2.1	9.4	**4.3**	

flhA		186	798	**358**	157	773	**90**	2.2	8.6	**4**	
fliF		181	729	**340**	266	994	**88**	2.1	11.3	**3.9**	
fliG		119	501	**202**	202	758	**58**	2.5	13	**3.5**	
FlgN		198	639	**165**	222	571	**40**	3.9	14.3	**4.1**	flagella synthesis protein
flgF		178	594	**266**	243	885	**77**	2.2	11.5	**3.4**	
**carbohydrate metabolism**											
cpsB		640	1545	**498**	625	1503	**36**	3.1	41.8	**13.8**	mannose-1-phosphate guanylyltransferase
manB		382	1005	**340**	437	1006	**20**	3	50.6	**17.1**	phosphomannomutase
wcaJ		163	378	**132**	269	640	**22**	2.9	29.3	**6**	UDP-glucose:undecaprenyl-phosphate glucose-1-phosphate transferase
manA_1		608	630	**299**	665	587	**30**	2.1	19.4	**9.9**	
gmd		718	1230	**131**	402	989	**38**	9.4	25.7	**3.4**	GDP-mannose 4,6-dehydratase
**envelope/membrane/cell division**											
phoB		229	307	**145**	151	192	**23**	2.1	8.2	**6.2**	
mepM		342	373	**176**	423	315	**56**	2.1	5.7	**3.2**	Peptidoglycan-specific endopeptidase
**others**											
**VC_2654**		324	379	**117**	244	165	**27**	3.2	6.1	**4.3**	rhodanese-like domain-containing protein thiosulfate disproportionation
aspA		251	488	**197**	358	559	**44**	2.5	12.7	**4.5**	
**gene name**		T0 ravvia	T16 ravvia	**T16 TOB ravvia**	T0 WT	T16 WT	**T16 TOB WT**	fold increase ravvia T16TOB/T16MH	fold increase WT T16TOB/T16MH	**FC T16 TOB wt/ravvia ****	
**TOB 16gen: insertions increase in wt but unchanged in ravvia => important for Δravvia growth in TOB or inactivation has no additional benefit in Δravvia => partners/linked processes?**											
**translation/protein stress**											
**VC_0803**	*trmH**	130	87	**148**	221	147	**801**	1.7	5.5	**5.4**	tRNA modification
	*rluB**	110	60	**96**	152	111	**1049**	1.6	9.5	**11.0**	rRNA modification
	*dusB**	183	118	**184**	116	91	**1118**	1.6	12.3	**6.1**	tRNA modification
	slyD	44	48	**75**	97	108	**348**	1.6	3.2	**4.7**	**Chaperone**
**iron**											
	hemF	108	95	**136**	87	104	**422**	1.4	4	**3.1**	
	*fur**	5	7	**7**	43	6	**157**	0.9	26.8	**23.1**	
**electron transport/ Fe-S/redox**											
	ccoO	115	75	**141**	205	47	**1405**	1.9	29.9	**9.9**	cytochrome C oxidase
	ccoG	163	81	**179**	181	124	**1938**	2.2	15.6	**10.8**	cytochrome c
	VC_0574	88	51	**136**	165	112	**1613**	2.7	14.4	**11.9**	cytochrome b
	VC_0575	136	37	**146**	114	53	**1432**	3.9	27	**9.8**	cytochrome c
	VC_0168	118	63	**165**	82	58	**1859**	2.6	32.1	**11.3**	cytochrome b
	ccmG	94	61	**92**	92	28	**441**	1.5	15.8	**4.8**	cytochrome c biogenesis protein
	ccmE	61	36	**81**	190	108	**846**	2.2	7.8	**10.4**	cytochrome c
	ccmA	77	47	**105**	117	60	**483**	2.2	8.1	**4.6**	cytochrome c

	ccmI	59	55	**105**	109	119	**353**	1.9	3	**3.4**	cytochrome c biogenesis
	arcA_2	147	47	**57**	113	23	**289**	1.2	12.6	**5.1**	response regulator, regulates cadA
	arcB	195	64	**97**	193	41	**480**	1.5	11.6	**4.9**	response regulator, regulates cadA
	dsbD dipZ	118	77	**146**	136	89	**686**	1.9	7.7	**4.7**	thiol:disulfide interchange
	ychF	72	21	**57**	99	58	**737**	2.7	12.7	**12.9**	redox-responsive ATPase
	*cadA = ldcI**	95	39	**115**	98	109	**1491**	2.9	13.7	**13.0**	
	*iscR**	21	27	**61**	47	46	**845**	2.2	18.4	**13.9**	
**carbohydrate metabolism**											
	*crr**	32	16	**14**	156	23	**489**	0.9	21.3	**33.9**	glucose uptake
	ptsI	74	19	**56**	99	27	**867**	3	32.7	**15.5**	
	ptsH	50	41	**3**	79	78	**230**	0.1	2.9	**86.6**	phosphocarrier protein HPr
	pykF	42	16	**39**	82	32	**218**	2.4	6.8	**5.6**	pyruvate kinase
	rpiR	115	64	**103**	84	32	**497**	1.6	15.4	**4.8**	carbohydrate utilization regulator
	rpe	174	32	**62**	175	39	**536**	1.9	13.9	**8.6**	ribulose-phosphate 3-epimerase
**envelope/membrane/motility**											
	bamC	255	100	**174**	180	88	**555**	1.7	6.3	**3.2**	outer membrane protein assembly
	mioC	175	50	**161**	113	69	**827**	3.3	12.1	**5.1**	flavoprotein, cell division
	VC_1422	166	148	**98**	160	143	**662**	0.7	4.6	**6.8**	sodium:alanine symporter
	hdfR	73	66	**137**	137	139	**779**	2.1	5.6	**5.7**	negative regulator of flagella
**others**											
	luxO	126	102	**168**	146	108	**711**	1.7	6.6	**4.2**	QS
	citG	193	191	**191**	296	283	**633**	1	2.2	**3.3**	
**unknown**											
	VC_0124	98	36	**47**	180	178	**1016**	1.3	5.7	**21.7**	putative lipoprotein L
	VC_2498	61	34	**112**	183	126	**1508**	3.3	12	**13.4**	
	VC_A0039	72	33	**135**	130	155	**1833**	4.1	11.8	**13.6**	putative cobaltochelatase subunit CobN
**YfcZ**	VC_A0919	60	107	**68**	284	174	**260**	0.6	1.5	**3.8**	
**ybfF**	VC_2097	100	122	**160**	201	225	**518**	1.3	2.3	**3.2**	
	VC_A1061	106	64	**54**	129	261	**163**	0.8	0.6	**3.0**	
	VC_2476	46	67	**53**	126	189	**228**	0.8	1.2	**4.3**	

****: FC T16TOB, only values with adjusted pvalue<0.01 are shown**

Table S5.

Strain #	*genotype*	resistance	parental strain	construction	primers for gibson assembly or details for cloning
J085	*WT V. cholerae N16961 hapR+*	strep		lab collection	
K329	*WT V. cholerae N16961 hapR+ ΔlacZ*	strep	J085	laboratory collection	deletion of lacZ by conjugation J085*4850 and sucrose 15% excision
L555	*ΔravA-viaA::spec ΔlacZ*	spec	K329	pMP7 L571	p9344 digested with EcoRI and cloned into pMP7 EcoRI site
M093	*ΔravA-viaA::kan ΔlacZ*	frt::kan::frt	K329	pMP7 L911	yeiMN5/7 for up region and yeiMN6/8 for down region
O390	*ΔravA-viaA ΔlacZ*		M093	excision of *frt::kan::frt*	
O506	*ΔravA-viaA pGBts-ravA-viaA+*	spec 30°C		transfomation of thermosensitive low copy pGBts plasmid expressing *ravvia* into *ravvia* deleted strain.	operon ravA-viaA with its own promoter amplified with primers Pravaviamonteco and Opravaviavaleco and clones inside EcoRI of pGBts
M145	*WT::Pbla-ravA-viaA-extracopy OE+*	Cm5	J085	tn7 transposition pM060	Pbla-ravA-viaA cloned in XmaI into pMVM4 (=L950)
N542	*ΔravA::kan ΔlacZ*	frt::kan::frt	K329	pMP7 N624	yeiMN5/7 for up region and yeiN6/8 for down region
N601	*ΔravA ΔlacZ*	-	N542	excision of *frt::kan::frt*	
N544	*ΔviaA::kan ΔlacZ*	frt::kan::frt	K329	pMP7 N625	yeiM5/7 for up region and yeiMN6/8 for down region
N620	*ΔviaA ΔlacZ*	-	N544	excision of *frt::kan::frt*	
S490	*ΔcpxPAR::kan*	frt::kan::frt	K329	pMP7 S448	cpxPAR5/7 for up region and cpxAR6bis/8 for down region
Q080	*Δzra2 (ΔVC1315-1316) ΔlacZ*	frt::kan::frt	K329	pMP7 P679	VC1315-165/7 for up region and VC1315-166/8 for down region

R292	*Δzra2 (ΔVC1315-1316) ΔlacZ*	-	Q080	excision of *frt::kan::frt*	
S492	*Δzra2 (ΔVC1315-1316) ΔlacZ ΔcpxPAR::kan*	frt::kan::frt	R292	pMP7 S448	cpxPAR5/7 for up region and cpxAR6bis/8 for down region
S565	*ΔlacZ ΔravA-viaA ΔcpxPAR::kan*	frt::kan::frt	O390	pMP7 S448	cpxPAR5/7 for up region and cpxAR6bis/8 for down region
R496	*ΔlacZ ΔravA-viaA Δzra2::kan*	frt::kan::frt	O390	pMP7 P679	VC1315-165/7 for up region and VC1315-166/8 for down region
S186	*ΔlacZ ΔravA-viaA Δzra2*		R496	excision of *frt::kan::frt*	
S547	*ΔravA-viaA ΔcpxPAR::kan Δzra2 ΔlacZ*	frt::kan::frt	S186	pMP7 S448	cpxPAR5/7 for up region and cpxAR6bis/8 for down region
P640	*ΔlacZ Δbcp::kan*	frt::kan::frt	K329	pMP7 O650	VC2160bcp5/7 for up region and VC2160bcp6/8 for down region
P739	*ΔravA-viaA Δbcp::kan ΔlacZ*	frt::kan::frt	O390	pMP7 O650	VC2160bcp5/7 for up region and VC2160bcp6/8 for down region
Q900	*ΔlacZ ΔfrdA-D::kan*	frt::kan::frt	K329	pMP7 P358	frdAD5/7 for up region and frdAD6/8 for down region
U239	*ΔravA-viaA ΔlacZ ΔfrdA-D::kan*	frt::kan::frt	O390	pMP7 P358	frdAD5/7 for up region and frdAD6/8 for down region
Q695	*ΔlacZ Δsdh::kan*	frt::kan::frt	K329	pMP7 P680	VC2088-91sdh5/7 for up region and VC2088-91sdh6bis/8 for down region
Q079	*ΔravA-viaA ΔlacZ Δsdh::kan*	frt::kan::frt	O390	pMP7 P680	VC2088-91sdh5/7 for up region and VC2088-91sdh6bis/8 for down region
Q063	*ΔlacZ ΔfeoC::kan*	frt::kan::frt	K329	pMP7 P347	VC2076feoC5/7 for up region and VC2076feoC6/8 for down region
Q064	*ΔravA-viaA ΔlacZ ΔfeoC::kan*	frt::kan::frt	O390	pMP7 P347	VC2076feoC5/7 for up region and VC2076feoC6/8 for down region
Q101	*ΔlacZ ΔiscX::kan*	frt::kan::frt	K329	pMP7 P349	VC0754iscX5/7 for up region and VC0754iscX6/8 for down region

Q066	*ΔravA-viaA ΔlacZ ΔiscX::kan*	frt::kan::frt	O390	pMP7 P349	VC0754iscX5/7 for up region and VC0754iscX6/8 for down region
Q068	*ΔravA-viaA ΔlacZ ΔtrxC::kan*	frt::kan::frt	O390	pMP7 P352	VCA0752trxC5/7 for up region and VCA0752trxC6/8 for down region
Q077	*ΔlacZ ΔcspE::kan*	frt::kan::frt	K329	pMP7 P677	VCA0184capB5/7 for up region and VCA0184capB6/8 for down region
Q078	*ΔravA-viaA ΔlacZ ΔcspE::kan*	frt::kan::frt	O390	pMP7 P677	VCA0184capB5/7 for up region and VCA0184capB6/8 for down region
L168	*ΔlacZ ΔmiaB::kan*	frt::kan::frt	K329	pMP7 J744	Negro et al, mBio, 2019
O980	*ΔravA-viaA ΔlacZ ΔmiaB::kan*	frt::kan::frt	O390	pMP7 J744	Negro et al, mBio, 2019
Q081	*ΔlacZ Δcrp::spec*	spec	K329	pMP7 8348	Baharoglu et al, J Bact, 2012
Q083	*ΔravA-viaA ΔlacZ Δcrp::spec*	spec	O390	pMP7 8348	Baharoglu et al, J Bact, 2012
Q070	*cpxP::kan ΔlacZ*	frt::kan::frt	K329	pMP7 P353	VC2691cpxP5/7 for up region and VC2691cpxP6/8 for down region
Q072	*ΔravA-viaA ΔcpxP::kan ΔlacZ*	frt::kan::frt	O390	pMP7 P353	VC2691cpxP5/7 for up region and VC2691cpxP6/8 for down region
S384	*ΔtatABC::kan ΔlacZ*	frt::kan::frt	K329	pMP7 F164	Krin et al, BMC , 2022
T618	*ΔravA-viaA ΔtatABC::kan ΔlacZ*	frt::kan::frt	O390	pMP7 F164	Krin et al, BMC , 2022
T654	*ΔlacZ ΔsgrR::kan*	frt::kan::frt	K329	pMP7 P676	VCA0578sgrR5bis/7 for up region and VCA0578sgrR6/8bis for down region
Q076	*ΔlacZ ΔravA-viaA ΔsgrR::kan*	frt::kan::frt	O390	pMP7 P676	VCA0578sgrR5bis/7 for up region and VCA0578sgrR6/8bis for down region
P645	*ΔlacZ Δrdx::kan*	frt::kan::frt	K329	pMP7 P331	VC0982rdx5/7 for up region and VC0982rdx6/8 for down region
P736	*ΔlacZ ΔravA-viaA Δrdx::kan*	frt::kan::frt	O390	pMP7 P331	VC0982rdx5/7 for up region and VC0982rdx6/8 for down region
Q061	*ΔlacZ ΔrluE::kan*	frt::kan::frt	K329		Babosan et al, microLife, 2022

T608	*ΔlacZ ΔravA-viaA ΔrluE::kan*	frt::kan::frt	O390	pMP7 P346	Babosan et al, microLife, 2022
P725	*ΔlacZ Δcrr::kan*	frt::kan::frt	K329	pMP7 P333	VC0964crr5/7 for up region and VC0964crr6/8 for down region
P744	*ΔravA-viaA ΔlacZ Δcrr::kan*	frt::kan::frt	O390	pMP7 P333	VC0964crr5/7 for up region and VC0964crr6/8 for down region
S388	*ΔlacZ ΔdcuA::kan*	frt::kan::frt	K329	pMP7 S156	dcuA5/7 for up region and dcuA6/8 for down region
T297	*ΔlacZ ΔdcuA*		S388	excision of *frt::kan::frt*	
S405	*ΔravA-viaA ΔlacZ ΔdcuA::kan*	frt::kan::frt	O390	pMP7 S156	dcuA5/7 for up region and dcuA6/8 for down region
T344	*ΔravA-viaA ΔlacZ ΔdcuA*		S405	excision of *frt::kan::frt*	
U352	*ΔlacZ ΔdcuB::kan*	frt::kan::frt	K329	pMP7 S158	dcuB5/7 for up region and dcuB6/8 for down region
U354	*ΔravA-viaA ΔlacZ ΔdcuB::kan*	frt::kan::frt	O390	pMP7 S158	dcuB5/7 for up region and dcuB6/8 for down region
U353	*ΔlacZ ΔdcuA dcuB::kan*	frt::kan::frt	T297	pMP7 S158	dcuB5/7 for up region and dcuB6/8 for down region
U356	*ΔravA-viaA ΔlacZ ΔdcuA dcuB::kan*	frt::kan::frt	T344	pMP7 S158	dcuB5/7 for up region and dcuB6/8 for down region
L559	*ΔlacZ ΔrluB::kan*	frt::kan::frt	K329		Babosan et al, microLife, 2022
T612	*ΔravA-viaA ΔlacZ ΔrluB::kan*	frt::kan::frt	O390	pMP7 L020	Babosan et al, microLife, 2022
L606	*ΔlacZ ΔdusB::kan*	frt::kan::frt	K329		Babosan et al, microLife, 2022
T614	*ΔravA-viaA ΔlacZ ΔdusB::kan*	frt::kan::frt	O390	pMP7 L416	Babosan et al, microLife, 2022
N540	*Δfur::kan*	frt::kan::frt	J085	pMP7 N231	VC2106fur5/7 for up region and VC2106fur9/8 for down region
Q896	*ΔravA-viaA ΔlacZ Δfur::kan*	frt::kan::frt	O390	pMP7 N231	VC2106fur5/7 for up region and VC2106fur9/8 for down region
Q699	*ΔlacZ ΔldcI::kan*	frt::kan::frt	K329	pMP7 P338	VC0281ldcIcada5/7 for up region and VC0281ldcIcada6/8 for down region

Q074	*ΔravA-viaA ΔlacZ ΔldcI::kan*	frt::kan::frt	O390	pMP7 P338	VC0281ldcIcada5/7 for up region and VC0281ldcIcada6/8 for down region
Q701	*ΔlacZ ΔiscR::kan*	frt::kan::frt	K329	pMP7 P355	VC0747iscR5/7 for up region and VC0747iscR6/8 for down region
U426	*ΔlacZ ΔiscR*		Q701	excision of *frt::kan::frt*	
Q899	*ΔlacZ ΔravA-viaA ΔiscR::kan*	frt::kan::frt	O390	pMP7 P355	VC0747iscR5/7 for up region and VC0747iscR6/8 for down region
U493	*ΔlacZ ΔravA-viaA ΔiscR*		Q899	excision of *frt::kan::frt*	
Q696	*ΔlacZ ΔptsI-H::kan*	frt::kan::frt	K329	pMP7 P494	VC0965-6ptsIH5/7 for up region and VCVC0965-6ptsIH6bis/8 for down region
S407	*ΔlacZ ΔravA-viaA ΔptsI-H::kan*	frt::kan::frt	O390	pMP7 P494	VC0965-6ptsIH5/7 for up region and VCVC0965-6ptsIH6bis/8 for down region
P638	*ΔlacZ ΔtruC::kan*	frt::kan::frt	K329		Babosan et al, microLife, 2022
P741	*ΔlacZ ΔravA-viaA ΔtruC::kan*	frt::kan::frt	O390	pMP7 O651	Babosan et al, microLife, 2022

Table S6: Primers

primers	
yeiMN5	CTATTATTTAAACTCTTTCCACGACAATCTCGCCTTGGT
yeiMN7	CTACACAATCGCTCAAGACGTGCTCTTGATTCCTCAGACAAAG
yeiMN8	CTAATTCCCATGTCAGCCGTCTTGAATGCTTCATACCCAAC
yeiMN6	TACGTAGAATGTATCAGACTATAAAAAAACGTCATAAGAATAGC
VC2106fur5	CTATTATTTAAACTCTTTCCAAGCGGATGCGAACTTCGC
VC2106fur7	CTACACAATCGCTCAAGACGTGATACTTTCCTGTTGATGTTCTGC
VC2106fur8	CTAATTCCCATGTCAGCCGTGCTCACAAGCCGAAGAAATAA
VC2106fur9	TACGTAGAATGTATCAGACTCCACAAATCGATCAGTTTATGG
yeiN8	CTAATTCCCATGTCAGCCGTGCATTCGTAACCTCAACCAA
yeiN6	TACGTAGAATGTATCAGACTACGCTCTTTGTTGGCTTTAAG
yeiM5	CTATTATTTAAACTCTTTCCCGTTATTGCAGAGCAATATGTC
yeiM7	CTACACAATCGCTCAAGACGTGAATGACACCTAAGCAAAAAATTG
Pravaviamonteco	GGAATTCTATTGAAACTATTGTTTATAGAGCG
Opravaviavaleco	GGAATTCTTACCACTTCTTCATTAGCCG
VCA0578sgrR5bis	CTATTATTTAAACTCTTTCCGACACGACAATCGCGTTACC
VCA0578sgrR7	CTACACAATCGCTCAAGACGTGAAAGAGGAAATCTCATCTAACTT
VCA0578sgrR8	CTAATTCCCATGTCAGCCGTCTTTTACTCACTCGTGGGAT
VCA0578sgrR6bis	TACGTAGAATGTATCAGACTTTGTTCTCTGCCATCTCTTTTTTC
VC0982rdx5	CTATTATTTAAACTCTTTCCATATTGGGGGAGTGACTTCA
VC0982rdx7	CTACACAATCGCTCAAGACGTGCGTGACGTCCTTGTTATGTC
VC0982rdx8	CTAATTCCCATGTCAGCCGTGCGATCCCCAAACGACTCAG
VC0982rdx6	TACGTAGAATGTATCAGACTAATCCTATCCCGCAAGGGTA
VC2160bcp5	CTATTATTTAAACTCTTTCCGAAGTGGTTCGATTAGTGAC
VC2160bcp7	CTACACAATCGCTCAAGACGTGAATTATCCCTTTGATTACTGACT
VC2160bcp8	CTAATTCCCATGTCAGCCGTATCGGTAGAAATGCCGATTTT
VC2160bcp6	TACGTAGAATGTATCAGACTGCCGATGAAGTTCGGACGTT
VC0964crr5	CTATTATTTAAACTCTTTCCAAGAAGTCTCTTTCTTCTCTATC
VC0964crr7	CTACACAATCGCTCAAGACGTGTGTCATGCTCCTAACGTT
VC0964crr8	CTAATTCCCATGTCAGCCGTGACCAAGTAATCGCTTGG

VC0964crr6	TACGTAGAATGTATCAGACTATTAAAGTGTTGCAACACGG
VC0965-6ptsIH5	CTATTATTTAAACTCTTTCCAAATGGCGCGTCGCCTAATG
VC0965-6ptsIH7	CTACACAATCGCTCAAGACGTGTTTTATACCCCAATGAGTTTA
VC0965-6ptsIH8	CTAATTCCCATGTCAGCCGTTATCGGTTGATACCAAGGA
VC0965-6ptsIH6bis	TACGTAGAATGTATCAGACTCAACGATTTTCTCAGCGAAAA
VC2076feoC5	CTATTATTTAAACTCTTTCCAGCACACCTGATGCAGAAGA
VC2076feoC7	CTACACAATCGCTCAAGACGTGCGACAGATACCTCTATCAT
VC2076feoC8	CTAATTCCCATGTCAGCCGTAACCTTCCAACTTGAAGGTG
VC2076feoC6	TACGTAGAATGTATCAGACTAGCTGGTTTGCCAAACTCTG
VC2691cpxP5	CTATTATTTAAACTCTTTCCGATCGCTAAAAGGTTTGGGC
VC2691cpxP7	CTACACAATCGCTCAAGACGTGCGTTCGTTCCTCTACATTTC
VC2691cpxP8	CTAATTCCCATGTCAGCCGTCAAAAAACACGCTAGTCAATAA
VC2691cpxP6	TACGTAGAATGTATCAGACTGTTGAGGATCAAAAAGCACG
VC0747iscR5	CTATTATTTAAACTCTTTCCAACGTACTGGCTTGACCAAT
VC0747iscR7	CTACACAATCGCTCAAGACGTGAATCACACCGTATCCACACT
VC0747iscR8	CTAATTCCCATGTCAGCCGTCGGCAAGGTTTACACTGGAG
VC0747iscR6	TACGTAGAATGTATCAGACTGCCGATTTCATTGTTCACGT
VC2088-91sdh5	CTATTATTTAAACTCTTTCCTAAAGTACGAACGTCAATCAC
VC2088-91sdh7	CTACACAATCGCTCAAGACGTGTCAGCTCCATTGAGCATTTAT
VC2088-91sdh8	CTAATTCCCATGTCAGCCGTACATATTAAGTCCATGTTGATC
VC2088-91sdh6bis	TACGTAGAATGTATCAGACTATTCGTTGGGTGCGCAGGCA
VC0754iscX5	CTATTATTTAAACTCTTTCCTATAAAAGACACAGACAAAGCG
VC0754iscX7	CTACACAATCGCTCAAGACGTGGTTAGCCTTCCTATTGGTT
VC0754iscX8	CTAATTCCCATGTCAGCCGTTCATCTCATGCAAAATGAAGTG
VC0754iscX6	TACGTAGAATGTATCAGACTGCCGCTAAAGCTTTCCACTC
VCA0752trxC5	CTATTATTTAAACTCTTTCCGCGTTGAGATCAAAGGCGGC
VCA0752trxC7	CTACACAATCGCTCAAGACGTGACTTCCTCTCTTTATTTTGCG
VCA0752trxC8	CTAATTCCCATGTCAGCCGTAACCAAGCGCTGACTAAATAA
VCA0752trxC6	TACGTAGAATGTATCAGACTACGTGCCAAAAGCCAAACAC
VC0281ldcIcada5	CTATTATTTAAACTCTTTCCAACGGTGTACCGAAAAAAG
VC0281ldcIcada7	CTACACAATCGCTCAAGACGTGTTGGACATCTCCAAGGCGAA
VC0281ldcIcada8	CTAATTCCCATGTCAGCCGTGCCAGCTTTACCCCATAAAG

VC0281ldcIcada6	TACGTAGAATGTATCAGACTCCCTCGCCACTCGCACCCAA
VC1315-165	CTATTATTTAAACTCTTTCCTTTATGTCACACACCGCGAC
VC1315-167	CTACACAATCGCTCAAGACGTGAACGTTTCCTTAATGGCCGA
VC1315-168	CTAATTCCCATGTCAGCCGTGCGATGTTAGGTGAGCTTAG
VC1315-166	TACGTAGAATGTATCAGACTACTAATGAGGGGCATGTTTAT
cpxPAR5	CTATTATTTAAACTCTTTCCGTTGAGGATCAAAAAGCACG
cpxPAR7	CTACACAATCGCTCAAGACGTGCAAAAAACACGCTAGTCAATAA
cpxAR8	CTAATTCCCATGTCAGCCGTAGCGCTTAACGCAGTAACTC
cpxAR6bis	TACGTAGAATGTATCAGACTCGCTCAGTGGCCTATCTT
VCA0184capB5	CTATTATTTAAACTCTTTCCTTTTCGCCTTTTCCACTCGG
VCA0184capB7	CTACACAATCGCTCAAGACGTGAATAAATATCCTAAAAACATTTTTTAAC
VCA0184capB8	CTAATTCCCATGTCAGCCGTCTGAAAGCATCAAAGTTCTGTAA
VCA0184capB6	TACGTAGAATGTATCAGACTGCGACGCCCATGCAGGCTAT
